# Self-assembled RNA nanocarrier-mediated chemotherapy combined with molecular targeting in the treatment of esophageal squamous cell carcinoma

**DOI:** 10.1186/s12951-021-01135-5

**Published:** 2021-11-25

**Authors:** Xiang Li, Li Zhang, Xiamei Guo, Fei Xie, Cheng Shen, Yali Jun, Chao Luo, Longfei Liu, Xiaojuan Yu, Zhengwei Zhang, Qilong Wang, Yong Gao, Keping Xu

**Affiliations:** 1 The Comprehensive Cancer Centre, Department of Central Laboratory, The Affiliated Huaian No.1 People’s Hospital, Nanjing Medical University, Huai’an, 223300 China; 2 Department of Thoracic Surgery, The Affiliated Huaian No.1 People’s Hospital Nanjing Medical University, Huai’an, 223300 China; 3 The Comprehensive Cancer Centre, Department of Clinical Oncology, The Affiliated Huaian No.1 People’s Hospital, Nanjing Medical University, Huai’an, 223300 China; 4 Department of Pathology, The Affiliated Huaian No.1 People’s Hospital, Nanjing Medical University, Huai’an, China

**Keywords:** RNA nanoparticles, Esophageal squamous cell carcinoma, Target therapy, miR-375, EGFR aptamer

## Abstract

**Background:**

Esophageal cancer is the fifth most common cancer affecting men in China. The primary treatment options are surgery and traditional radio-chemotherapy; no effective targeted therapy exists yet. Self-assembled RNA nanocarriers are highly stable, easily functionally modified, and have weak off-tumor targeting effects. Thus, they are among the most preferred carriers for mediating the targeted delivery of anti-tumor drugs. miR-375 was found to be significantly down-regulated in esophageal squamous cell carcinoma (ESCC) tissues and its overexpression effectively inhibits the proliferation, migration, and invasion of ESCC cells. Moreover, epidermal growth factor receptor (EGFR) was overexpressed in ESCC cells, and accumulation of RNA nanoparticles in ESCC tumors was enhanced by EGFR-specific aptamer (EGFR_apt_) modification.

**Results:**

Herein, a novel four-way junction RNA nanocarrier, 4WJ-EGFR_apt_-miR-375-PTX simultaneously loaded with miR-375, PTX and decorated with EGFR_apt_, was developed. In vitro analysis demonstrated that 4WJ-EGFR_apt_-miR-375-PTX possesses strong thermal and pH stabilities. EGFR_apt_ decoration facilitated tumor cell endocytosis and promoted deep penetration into 3D-ESCC spheroids. Xenograft mouse model for ESCC confirmed that 4WJ-EGFR_apt_-miR-375-PTX was selectively distributed in tumor sites via EGFR_apt_-mediating active targeting and targeted co-delivery of miR-375 and PTX exhibited more effective therapeutic efficacy with low systemic toxicity.

**Conclusion:**

This strategy may provide a practical approach for targeted therapy of ESCC.

**Graphical Abstract:**

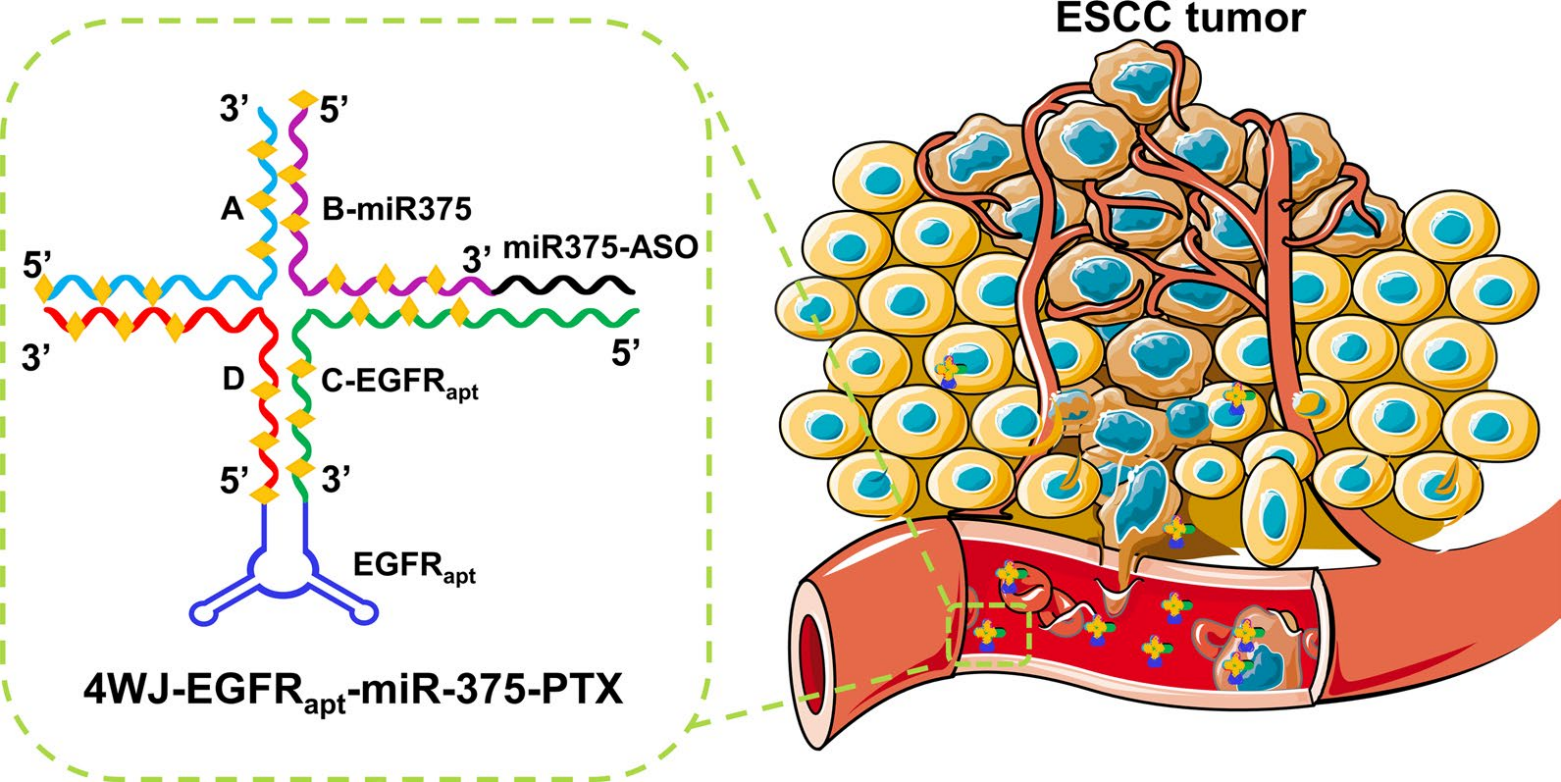

**Supplementary Information:**

The online version contains supplementary material available at 10.1186/s12951-021-01135-5.

## Background

The latest statistics showed that there were approximately 19.29 million new cases of cancer and 9.96 million new cancer-related deaths worldwide in 2020. Among these were 600,000 new cases and 548,000 deaths related to esophageal cancer [1]. The incidence and mortality of esophageal cancer in China rank first in the world, with the predominant subtype being squamous cell carcinoma, which accounts for more than 90% of the cases. Currently, the treatment of esophageal cancer remains a comprehensive approach based on surgery and traditional radio-chemotherapy, with chemotherapy drugs primarily used to treat advanced stages of cancer [2]. However, the lack of targeting ability of chemotherapeutic drugs and the serious side effects produced greatly limit their therapeutic effects. Therefore, current research is focused on developing new targeted treatment strategies against esophageal squamous cell carcinoma (ESCC).

In recent years, nanomedicine has played an increasingly important role in the precision diagnosis and treatment of tumors. The main advantages of this application include enhancing the carrier-mediated targeted distribution of drugs in tumors [3, 4], reducing the toxic and side effects of chemotherapy [5], and producing a more significant synergistic tumor inhibitory effect via combined drug delivery [6, 7]. Nevertheless, existing nanocarriers (such as inorganic nanomaterials [8], polymer nanomaterials [9], carbon nanomaterials [10], and cationic liposomes [11]) display a notably significant non-tumor tissue distribution effect, with a considerably lower level of drug accumulation at the tumor site than in organs and tissues such as the liver, kidney, and spleen. Owing to the low efficiency of targeted drug delivery, it is difficult to avoid toxic and side effects. Guo et al. revealed in 1998 that the packaging RNA (pRNA) of bacteriophage phi29 can be transformed—through self-assembly technology—into a dimer, trimer, or hexamer using a concise RNA structure [12]. Studies have confirmed that, in addition to possessing the characteristics of a strong enzyme [13], thermal stability [14, 15], and easy functional modification properties [16, 17], the RNA nanocarriers display a lower off-targeting effect than cationic liposomes due to their high degree of anionicity. When RNA nanocarriers are intravenously administered to subcutaneous xenotransplantation or metastatic tumor-bearing mice, they can specifically target cancer cells with little or no accumulation in normal organs or tissues, thereby exerting a better anti-tumor effect [13, 18, 19].

From our previous studies, we determined, through high-throughput sequencing, that miR-1973, miR-1246, and miR-375 were markedly under-expressed in ESCC tissues, and that their overexpression could significantly inhibit the proliferation, migration, and invasion of ESCC KYSE-150 cells. Among these, miR-375 was the most effective, highlighting its potential as a molecular target for the treatment of ESCC. Moreover, ESCC cells can overexpress the epidermal growth factor receptor (EGFR). The aptamer modification of EGFR (EGFR_apt_) can not only effectively increase the accumulation and tumor penetration ability of the RNA nanocarrier 4WJ in ESCC cells, but also significantly increase the distribution of carriers in mouse xenograft ESCC tissues. With these characteristics and the RNA four-way junction (4WJ) as a premise, the objective of the present study was to construct the aptamer-modified nano-drug 4WJ-EGFR_apt_-miR-375-PTX, which was simultaneously loaded with miR-375 and paclitaxel (PTX). We determined through experimental analyses that the nano-drug possesses strong enzyme and thermal stability. Cytological analysis of small animal ESCC tumor-bearing models have confirmed that the modification of EGFR_apt_, as well as the synergistic delivery of miR-375 and PTX, can exert a more effective inhibitory effect on ESCC, thus providing a potential strategy for targeted therapy against this cancer type.

## Results and discussion

### Detection and functional verification of miRNA

**Fig. 1:**
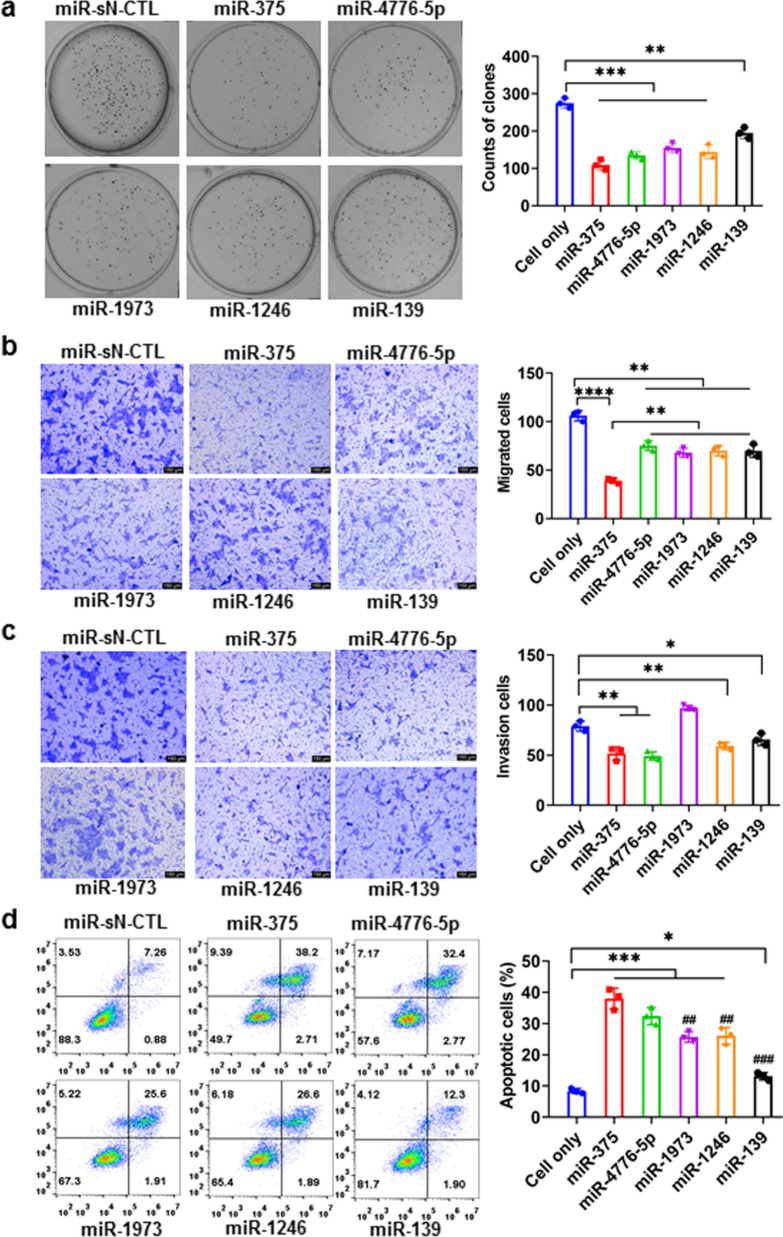
Effects of miRNAs on ESCC cells. **a** Colony-formation, **b** migration, **c** invasion, and **d** apoptosis of KYSE-150 cells after transfection with different miRNAs (miR-375, miR-4776-5p, miR-1973, miR-1246 and miR-139). *p < 0.05, **p < 0.01 and ***p < 0.001. Data are representative of at least three independent replicates from three independent experiments

MicroRNAs (miRNAs) are regulatory small non-coding RNAs of approximately 22 nt produced by virtually all the cells in the body [20]. They are important, highly conserved, non-coding small single-stranded RNAs, which play a key regulatory role in the occurrence and development of tumors [21, 22]. We analyzed the differential expression of miRNAs in 5-paired ESCC and normal esophageal tissues using high-throughput sequencing and observed that the expression of miR-1973, miR-1246, and miR-375 was significantly low in ESCC (Additional file 1: Fig. S1). The results of cytological analyses revealed that miR-375 was the most effective in inhibiting proliferation (Fig. 1a), migration (Fig. 1b), invasion (Fig. 1c), and in promoting apoptosis in ESCC cells (Fig. 1d), indicating miR-375 as a potential therapeutic target for ESCC. Currently, clinical trials involving certain miRNA analogs (e.g., miR-34 and miR-16) have produced positive results [23, 24], further highlighting the potential clinical application of miR-375.

### EGFR aptamer modification promotes the accumulation of RNA nanocarriers in esophageal squamous cell carcinoma cells and 3D tumor microspheres

Normal cells in the body undergo a series of pathological changes (e.g., gene mutations or changes in expression levels) during the process of canceration. For example, EGFR, a member of the epidermal growth factor receptor family, is overexpressed in several solid tumors, and its overexpression is closely related to tumor proliferation, angiogenesis, invasion, and metastasis [25]. Thus, EGFR is one of the preferred genes used in designing and developing tumor-targeted therapies such as cetuximab [26] and gefitinib [27]. By analyzing the TCGA database, we found that EGFR is highly expressed in ESCC (Additional file 1: Fig. S2), and this expression was further confirmed by testing 140 ESCC tissue samples (Additional file 1: Fig. S3a, b). KYSE-150 cells (Additional file 1: Fig. S4) also demonstrated a high expression of EGFR, indicating that EGFR can be used as a candidate in therapy against ESCC.

Therefore, we aimed to use EGFR as the candidate to construct a novel targeted nano-delivery system that is simultaneously loaded with miR-375 and chemotherapeutics, as a treatment against ESCC. The selection of suitable molecules that mediate targeting is the key to the construction of effective delivery systems. It is well-known that the ligands which mediate the targeted delivery of nanomedicine into tumors mainly include monoclonal antibodies [28], peptides [29], folic acid [30], and nucleic acid aptamers [31]. Aptamers are random oligonucleotide sequences that bind with high affinity and specificity to their target molecules, which are generated via screening using the systematic evolution of ligands by exponential enrichment (SELEX) technology. In addition to a high affinity and specificity comparable to those of antibodies, aptamers also have a wide range of targets, low molecular weight, low toxicity, non-immunogenicity, and high tissue permeability, contrary to antibodies [32]. They are currently one of the preferred candidates for mediating targeted nano-drug delivery. As mentioned previously, RNA nanoparticles have multiple advantages as drug delivery carriers. In the present study, the selected delivery carrier was 4WJ, which not only possesses the above characteristics but can also substantially increase the loading of chemotherapeutic drugs, as well as display higher thermodynamic stability than 3WJ [33]. Therefore, a EGFR_apt_-modified 4WJ carrier (4WJ-EGFR_apt_) was developed (Additional file 1: Fig. S5), and the data showed that EGFR_apt_ effectively promoted the accumulation of 4WJ in KYSE-150 cells (Additional file 1: Fig. S6a, b). The mouse ESCC tumor-bearing model also showed that EGFR_apt_ significantly promoted the distribution of 4WJ within the tumor site (Additional file 1: Fig. S7a, b), further indicating that EGFR_apt_ modification can mediate the targeted delivery of 4WJ-based nano-drugs to the ESCC sites.

### Construction and characterization of 4WJ-EGFR_apt_-miR-375-PTX

**Fig. 2:**
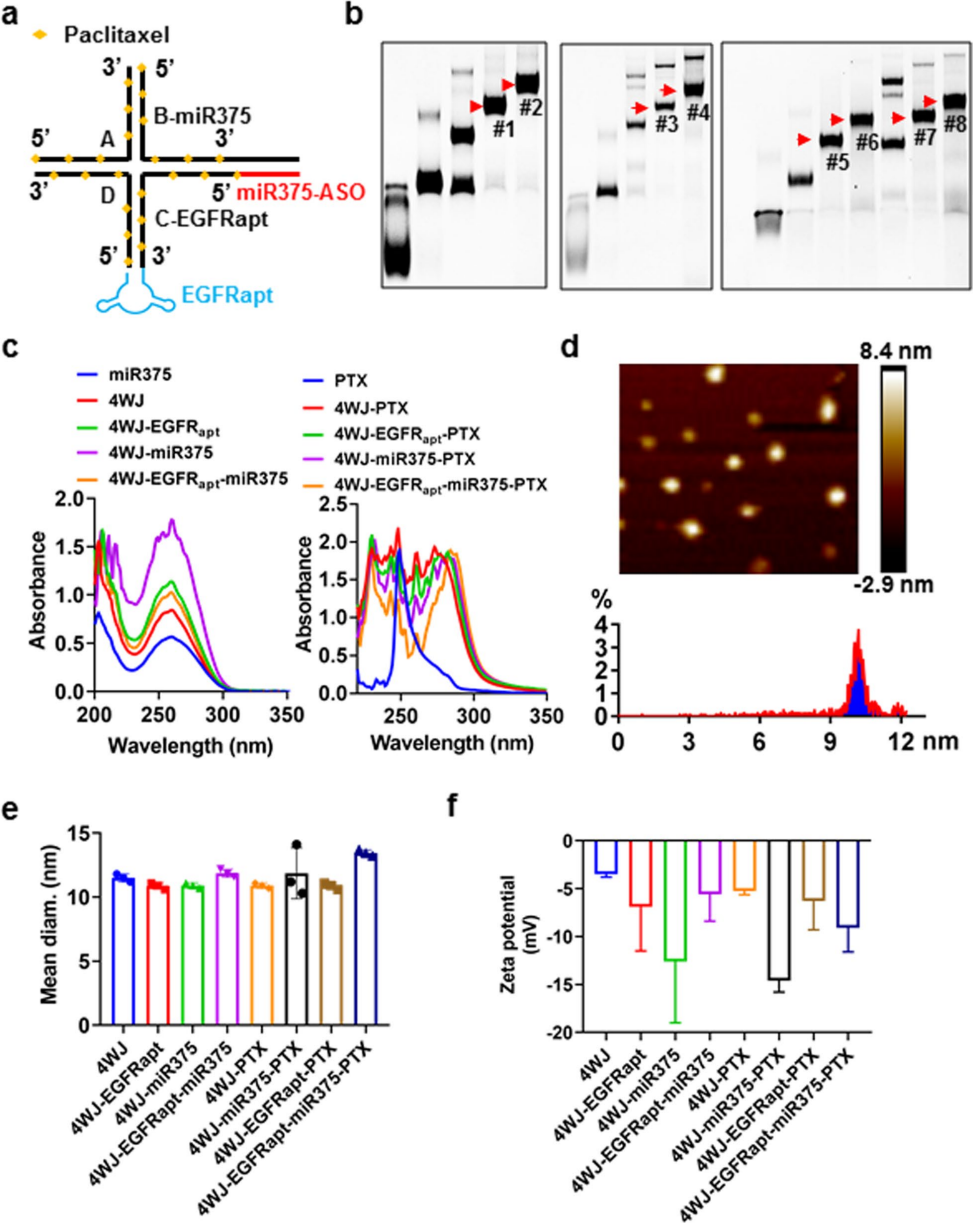
Construction and characterization of nanoparticles. **a** Schematic of 4WJ-EGFR_apt_-miR-375-PTX self-assembly. **b** Stepwise self-assembly of 4WJ (#1), 4WJ-miR-375 (#2), 4WJ-EGFR_apt_ (#3), 4WJ-EGFR_apt_-miR-375 (#4), 4WJ-PTX (#5), 4WJ-miR-375-PTX (#6), 4WJ-EGFR_apt_-PTX (#7) and 4WJ-EGFR_apt_-miR-375-PTX (#8). **c** Ultraviolet spectrum detection of nanoparticles. **d** Representative atomic force microscopy image of 4WJ-EGFR_apt_-miR-375-PTX. **e** Average size and **f** surface zeta potential of nanoparticles. Three independent samples over three independent measurements

To test whether 4WJ-EGFR_apt_ could mediate the targeted delivery of miR-375 and PTX, thereby achieving better therapeutic effects against ESCC, we constructed the EGFR_apt_-modified drug delivery system 4WJ-EGFR_apt_-miR-375-PTX, which was simultaneously loaded with miR-375 and 24 molecules of PTX (Fig. 2a). PTX-N_3_ was synthesized (Additional file 1: Fig. S8) and conjugated to different RNA oligomers (Additional file 1: Fig. S9). The nanoparticles were assembled by mixing equimolar concentrations of four RNA-6 PTX oligomers and preliminary identified by native PAGE (Fig. 2b) and UV absorption (Fig. 2c). The atomic force microscopy (AFM) images (Fig. 2d, Additional file 1: Fig. S10) and the results obtained from dynamic light scattering (DLS) showed the average particle size of the nanocarriers to be approximately 10 nm, whereas that of 4WJ-EGFR_apt_-miR-375-PTX was approximately 13.4 ± 0.25 nm (Fig. 2e). The zeta potential on the surface of all nanocarriers was negative, and that of 4WJ-EGFR_apt_-miR-375-PTX was about − 9.1 ± 2.5 mV (Fig. 2f). Surface anionicity is another key advantage of RNA nanocarriers that can effectively reduce the non-target cell-binding effect of nano-drugs.

### Stability of the nano-drug and its release from 4WJ-EGFR_apt_-miR-375-PTX

**Fig. 3:**
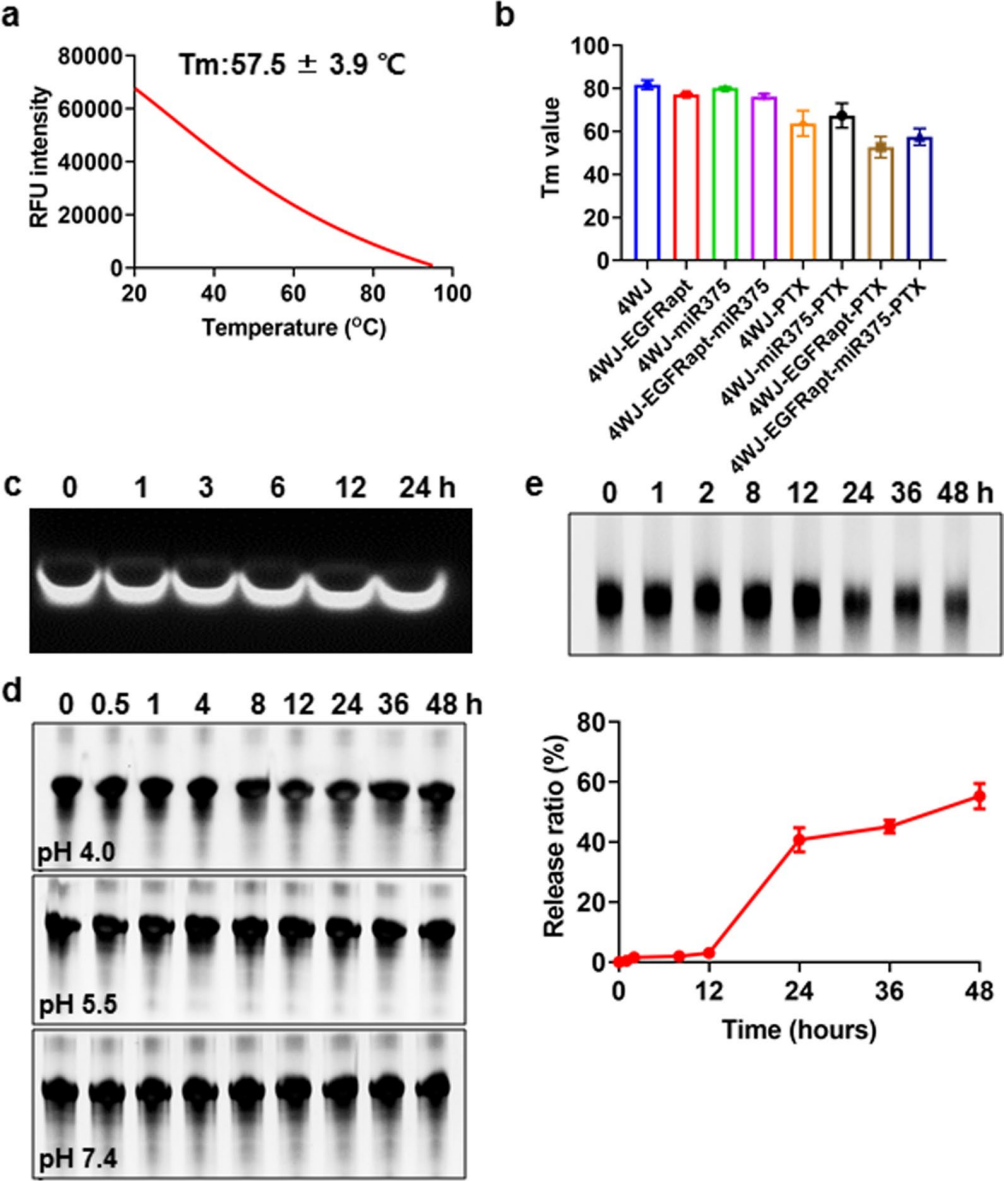
Thermodynamic, pH stability and PTX release profile of 4WJ-EGFR_apt_-miR-375-PTX. **a** Representative annealing curve of 4WJ-EGFR_apt_-miR-375-PTX measured by RT-PCR. **b** Tm values of 4WJ, 4WJ-EGFR_apt_, 4WJ-miR-375, 4WJ-EGFR_apt_-miR-375, 4WJ-PTX, 4WJ-EGFR_apt_-PTX, 4WJ-miR-375-PTX and 4WJ-EGFR_apt_-miR-375-PTX. **c** Enzymatic stability of 4WJ-EGFRapt-miR-375-PTX. **d** Stability of 4WJ-EGFR_apt_-miR-375-PTX in PBS with pH 4.0, 5.5 and 7.4 analyzed by native PAGE electrophoresis. **e** PTX release from 4WJ-EGFR_apt_-miR-375-PTX was validated by native PAGE electrophoresis. Data are representative of at least three independent experiments with three replicates

Once inside the body, the stability of the nanomedicine structure and—more importantly—rapid RNA degradation, are critical factors to achieve optimal pharmacokinetics and pharmacodynamics, as well as low toxic and side effects. However, studies have reported that RNA nanocarriers with special structures such as 4WJ possess superior enzyme and thermal stability. Moreover, the loading of the drug will also affect the stability of the carrier. For example, thermal stability is significantly reduced after loading the 3WJ carrier with 10 molecules of PTX [33]. Here, we tested the thermal, pH, and enzyme stability of 4WJ-EGFR_apt_-miR-375-PTX. The results revealed that 4WJ-EGFR_apt_-miR-375-PTX possessed good thermal stability and its melting temperature was 57.5 ± 3.9 °C (Fig. 3a). However, its stability was slightly lower than that of 4WJ, 4WJ-EGFR_apt_, 4WJ-miR-375, 4WJ-EGFR_apt_-miR-375, 4WJ-PTX, and 4WJ-EGFR_apt_-PTX (Fig. 3b, Additional file 1: Fig. S11). No significant degradation was observed after incubating 4WJ-EGFR_apt_-miR-375-PTX with RNase for up to 24 h (Fig. 3c). RNA nanoparticles of 4WJ in this study consists of fully modified RNA oligonucleotides at their 2’ position, e.g. 2’F, 2’OMe, which prevent them from RNase and plasma degradation, and this was identical with the previous report [34]. The stability of 4WJ-EGFR_apt_-miR-375-PTX in PBS with different pH was also tested, and the results suggested that the stability was not affected by pH variation (Fig. 3d). Drug release is another key factor that affects drug efficacy. A release assay showed that PTX was gradually released from 4WJ-EGFR_apt_-miR-375-PTX after incubation with 50% FBS, and significant release occurred after 12 h (Fig. 3e). The release of RNA nano-loaded PTX occurs mainly through the action of esterase which breaks the ester bonds.

### Inhibitory effect of 4WJ-EGFR_apt_-miR-375-PTX on esophageal squamous cell carcinoma cells in vitro

**Fig. 4:**
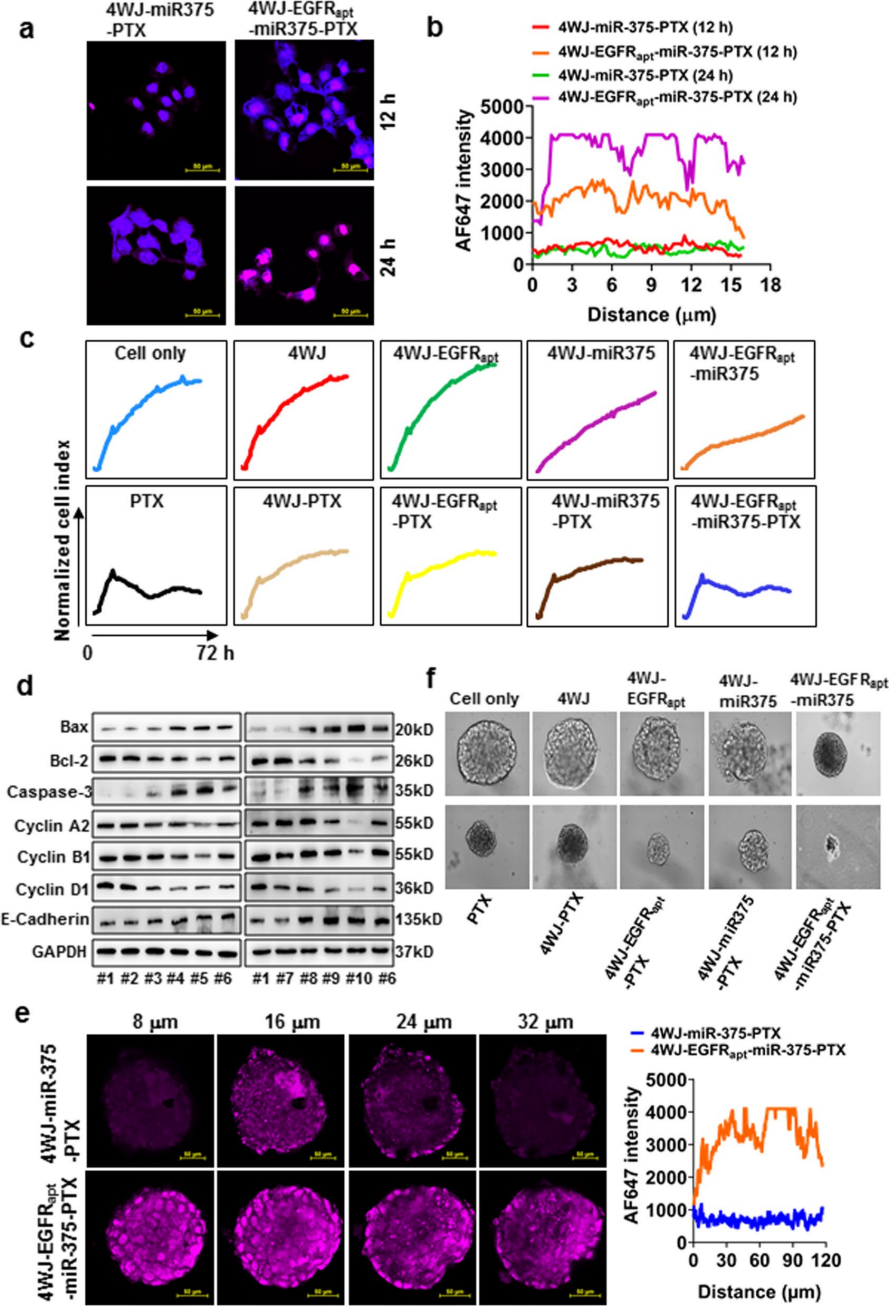
Uptake efficiency, tumor penetration and in vitro cytotoxicity analysis. **a** Uptake efficiency comparation between 4WJ-miR-375-PTX and 4WJ-EGFR_apt_-miR-375-PTX by KYSE-150 cells. **b** Quantify the nanoparticles in KYSE-150 cells. **c** Proliferation of KYSE-150 cells after treatment by 4WJ, 4WJ-EGFR_apt_, 4WJ-miR-375, 4WJ-EGFR_apt_-miR-375, PTX, 4WJ-PTX, 4WJ-EGFR_apt_-PTX, 4WJ-miR-375-PTX and 4WJ-EGFR_apt_-miR-375-PTX. **d** Western blotting of Bax, Bcl-2, caspase-3, Cyclin A2, Cyclin B1, Cyclin D1 and E-Cadherin in KYSE-150 cells treated with PBS (#1), 4WJ (#2), 4WJ-miR-375 (#3), 4WJ-PTX (#4), 4WJ-miR-375-PTX (#5), PTX (#6), 4WJ-EGFR_apt_ (#7), 4WJ-EGFR_apt_-miR-375 (#8), 4WJ-EGFR_apt_-PTX (#9) and 4WJ-EGFR_apt_-miR-375-PTX (#10). **e** Permeability comparation of 4WJ-miR-375-PTX and 4WJ-EGFR_apt_-miR-375-PTX in 3D tumor spheroids. **f** Inhibitory effects of PTX and nanodrugs on 3D tumor spheroids. Scale bar: 50 μm. Three independent replicates over at least three independent measurements

To test whether EGFR_apt_ can improve the efficiency of 4WJ-targeted delivery of miR-375 and PTX, we assessed the ability of EGFR_apt_-conjugated nanomedicine to bind and integrate KYSE-150 cells through cytological experiments. Confocal analysis revealed that EGFR_apt_ significantly enhanced the accumulation of 4WJ-miR-375-PTX in KYSE-150 cells (Fig. 4a, 4b). Cell proliferation assays showed that both 4WJ-miR-375 and 4WJ-EGFR_apt_-miR-375 inhibited the proliferation of KYSE-150 cells. 4WJ-PTX, 4WJ-EGFR_apt_-PTX, and 4WJ-miR-375-PTX significantly suppressed the proliferation of KYSE-150 cells; however, this inhibitory effect was weaker than those of PTX and 4WJ-EGFR_apt_-miR-375-PTX at low concentrations (120 nM PTX, 5 nM nanoparticles) (Fig. 4c). This inhibitory effect tended to be consistent with PTX and 4WJ-EGFR_apt_-miR-375-PTX as their concentrations increased (Additional file 1: Fig. S12). This may be related to the cell uptake and drug release kinetics. Studies have demonstrated that PTX exert the anti-tumor efficiency by inducing apoptosis and suppressing proliferation, migration and invasion. To elucidate the potential mechanism of synergetic effect of miR-375 and PTX on ESCC inhibition, apoptosis (Bax, Bcl2 and caspase-3), cell cycle (Cyclin A2, Cyclin B1 and Cyclin D1), migration and invasion-related proteins (E-cadherin) were determined. Data (Fig. 4d) suggested that the improved anti-ESCC effect of 4WJ-EGFR_apt_-miR-375-PTX is due to apoptosis, cell cycle and epithelial-mesenchymal transition arrest induced by miR-375.

Anti-tumor drugs must reach the tumor site and penetrate the tissue to exert their inhibitory effects. However, the features of tumor microenvironment, which include high peritumoral and low intratumoral blood vessel density, and a dense interstitial structure in the center of the tumor, lead to poor permeability of nanomedicines [35]. Most of the nanocarriers, therefore, are unable to consistently deliver the drug throughout the entire tumor tissue. This eventually results in uneven drug distribution, and, consequently, poor therapeutic efficacy. Therefore, enhancing the tumor permeability has become one of the most important strategies to improve the therapeutic efficacy of nanodrugs. Studies have confirmed that aptamers exhibit strong tissue permeability and their modification can significantly enhance the penetration of nanoparticles into tumors [36, 37]. In the present study, 3D-tumor spheroids of KYSE-150 cells were established and confocal analysis confirmed that the permeability of 4WJ-miR-375-PTX was dramatically enhanced by EGFR_apt_ decoration (Fig. 4e), and thus, 4WJ-miR-375-PTX exerted the strongest inhibitory effect on the growth of spheroids (Fig. 4f).

### In vivo biodistribution and anti-esophageal squamous cell carcinoma activity of 4WJ-EGFR_apt_-miR-375-PTX

**Fig. 5:**
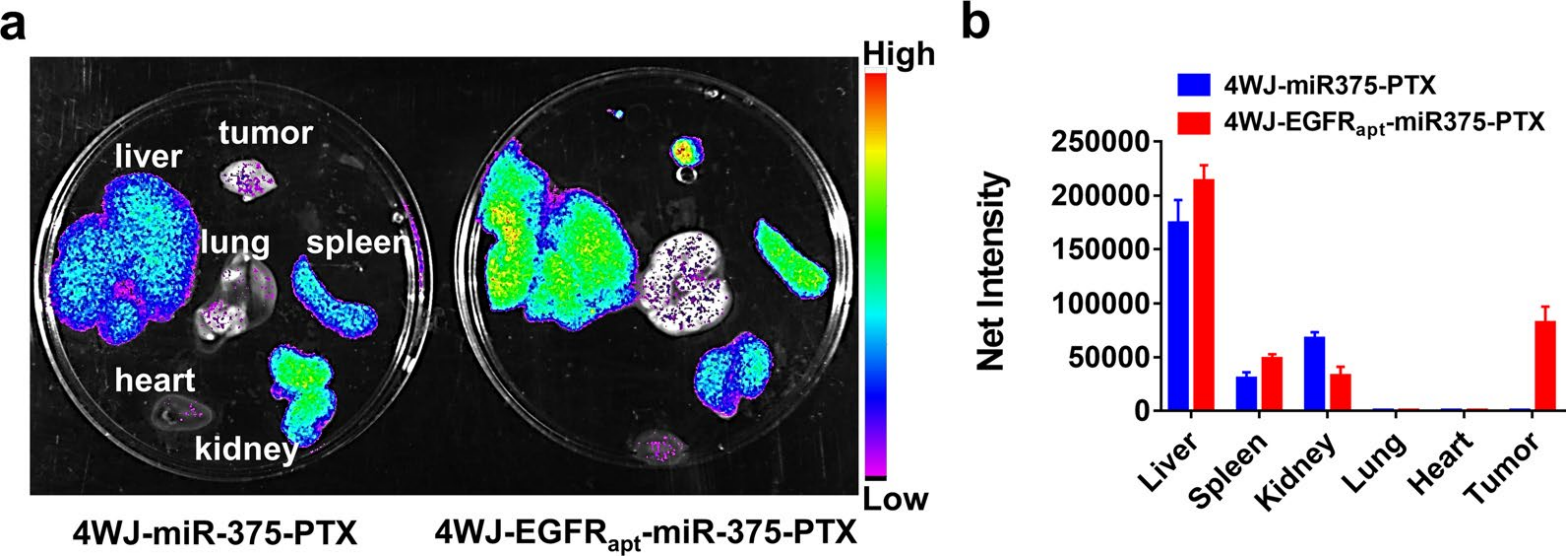
Biodistribution of 4WJ-miR-375-PTX and 4WJ-EGFR_apt_-miR-375-PTX. **a** AF647 labeled 4WJ-miR-375-PTX and 4WJ-EGFR_apt_-miR-375-PTX were respectively injected into ESCC tumor mice, the distribution in major organs was scanned by live imaging and **b** quantified. Data are representative of three independent experiments with 3 mice per group

The above data (Additional file 1: Fig. S7a, b) have confirmed that EGFR_apt_ modification can promote the targeted distribution of 4WJ in ESCC tissues. Therefore, we further investigated whether miR-375 and PTX affected the biodistribution of the nanoparticles. 4WJ-miR-375-PTX and 4WJ-EGFR_apt_-miR-375-PTX were separately injected intravenously into KYSE-150 tumor-bearing mice. Live imaging revealed that the distribution of 4WJ-EGFR_apt_-miR-375-PTX in the tumor tissues was much higher than that of 4WJ-miR-375-PTX (Fig. 5a, b), which supports the application of 4WJ-EGFR_apt_-miR-375-PTX in ESCC treatment.

**Fig. 6:**
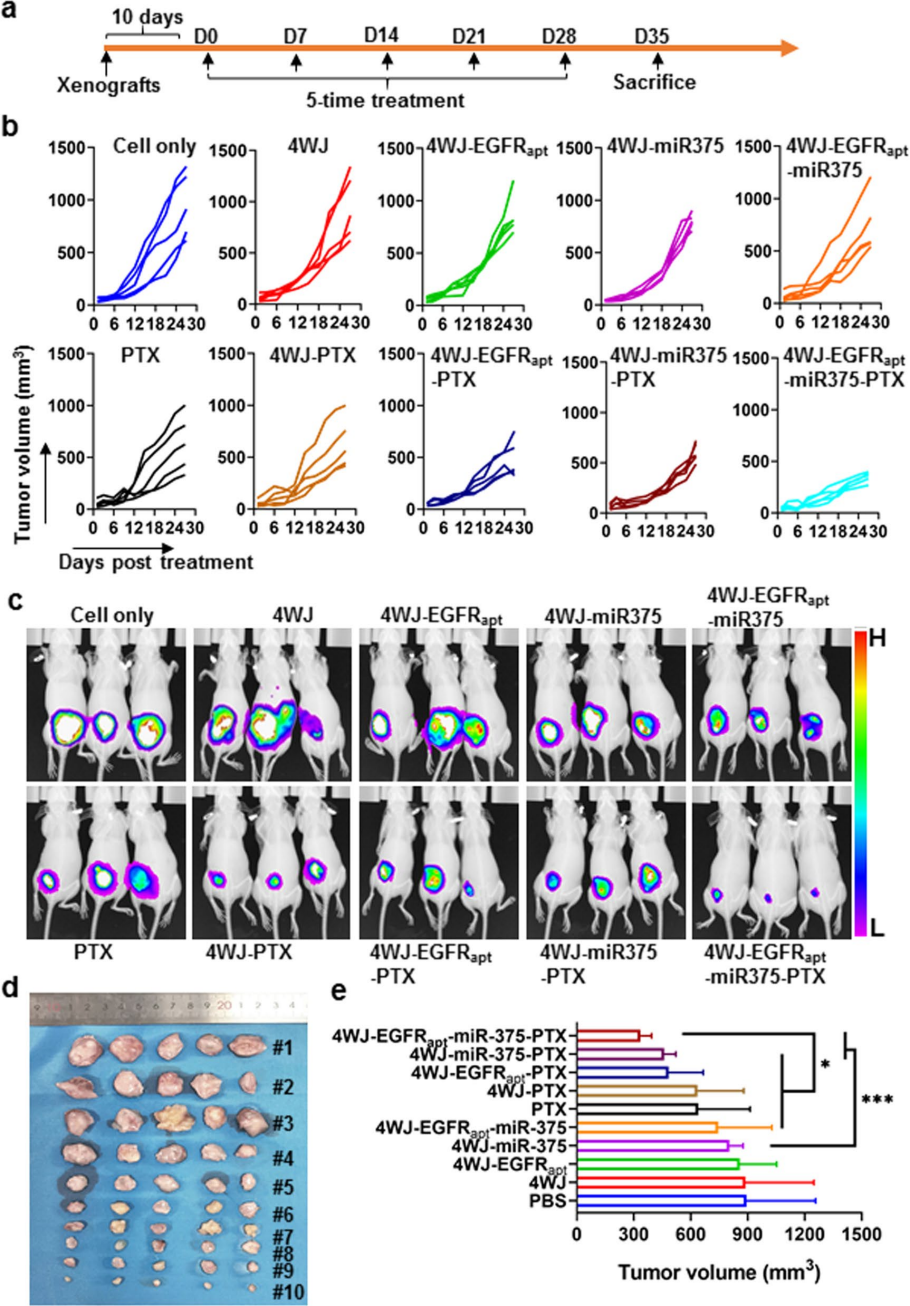
Antitumor efficacy of nanodrugs and PTX on ESCC xenograft mice. **a** Schematic of the treatment protocol of KYSE-150 tumor-bearing mice with nanoparticles and PTX. Tumor growth was monitored by volume measuring **b** and luciferase signal detection **c**. **d** Mice were sacrificed and tumors were photographed and volume was measured. *p < 0.05 and ***p < 0.001. Data are representative of three independent experiments with 5 mice per group

Next, mouse xenograft model of ESCC was established, and the mice were separately treated with PBS, 4WJ, 4WJ-EGFR_apt_, 4WJ-miR-375, 4WJ-EGFR_apt_-miR-375, PTX, 4WJ-PTX, 4WJ-EGFR_apt_-PTX, 4WJ-miR-375-PTX, and 4WJ-EGFR_apt_-miR-375-PTX administered intravenously every 7 days for a total of 5 times (Fig. 6a). Tumor volume measurement (Fig. 6b) and luciferase signal detection (Fig. 6c) indicated that the inhibitory effects of 4WJ-miR-375, 4WJ-PTX, and 4WJ-miR-375-PTX on KYSE-150 cells were significantly enhanced by EGFR_apt_ modification (Fig. 6b, c). Although 4WJ-miR-375-PTX was not EGFR_apt_-modified, its inhibitory effect was similar to that of 4WJ-EGFR_apt_-PTX and may be related to the synergistic effect of miR-375 and PTX. Seven days after the last treatment, the mice were sacrificed and the tumors were removed. The results of tumor imaging (Fig. 6d) and volume measurement (Fig. 6e, *p < 0.05, ***p < 0.001) were consistent with the above results. Cell proliferation in tumor tissues further confirmed that 4WJ-EGFR_apt_-miR-375-PTX exhibited the strongest anti-KYSE-150 effect in vivo (Additional file 1: Fig. S13). These results collectively indicated that EGFR_apt_-mediated targeted co-delivery of miR-375 and PTX using 4WJ can produce a more effective treatment against ESCC in vivo.

### In vivo toxicity analysis

**Fig. 7:**
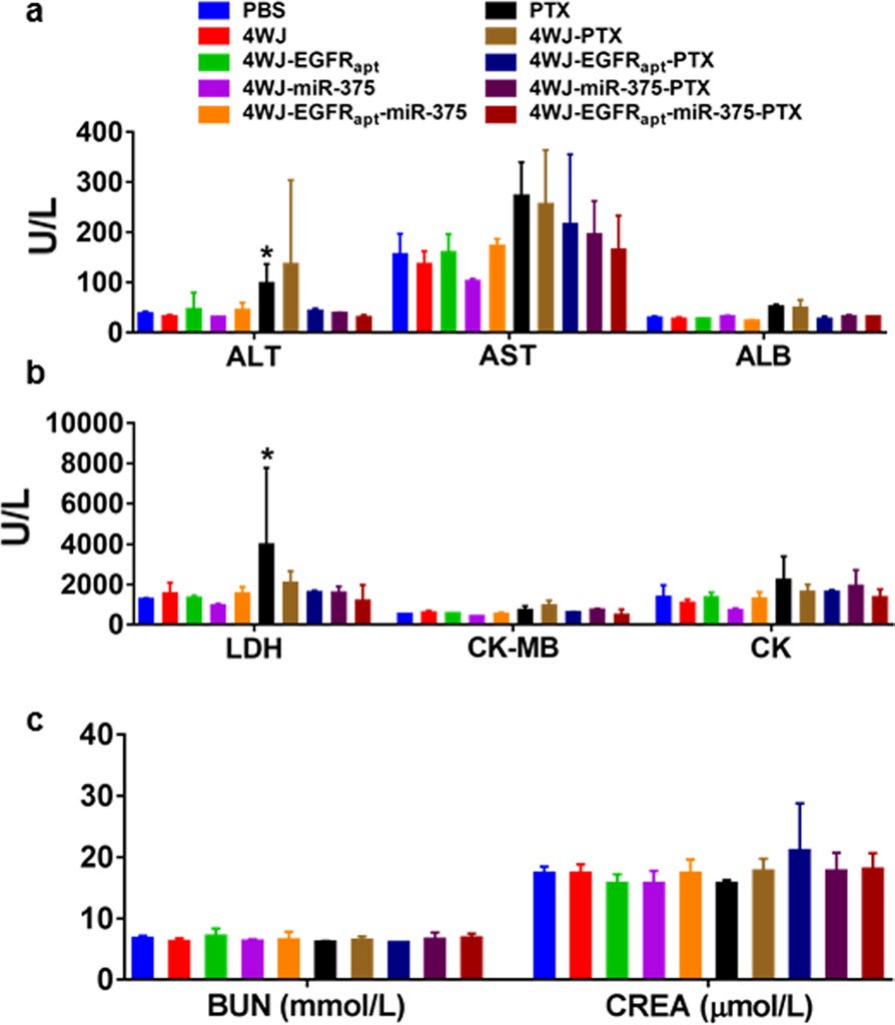
In vivo toxicity analysis. Peripheral blood was collected and the blood routine examination was carried out to detect the biomarkers for the **a** liver (ALT, AST and ALB), **b** heart (LDH, CK-MB and CK), and c) kidney (BUN and UREA). *p < 0.05. Five independent samples over three independent measurements

Potential toxic and side effects are important indicators to evaluate the application value of drugs in clinical transformation. PTX is one of the most widely used chemotherapeutic agents and displays a positive therapeutic effect during the treatment of ESCC. However, in its conventional formulation, PTX would cause strong toxic and side effects, including hypersensitivity reactions, myelosuppression, neurotoxicity, cardiotoxicity, hepatotoxicity, and alopecia [38]. We analyzed the following changes in mice treated five times with PTX and other nano-drugs: body weight, histopathology, blood biochemical indicators (hepatotoxicity indicators such as aspartate transaminase (AST), alanine transaminase (ALT), and albumin (ALB); kidney toxicity indicators such as blood urea nitrogen (BUN), and creatinine (CREA); and cardiotoxicity indicators such as lactate dehydrogenase (LDH), creatine kinase (CK), creatine kinase myocardial band (CK-MB)). Although no significant change was recorded in the body weight (Additional file 1: Fig. S14) and histopathology (Additional file 1: Fig. S15) of the mice in each group, analysis of the biochemical indicators showed that the hepatotoxicity indicator (ALT) (Fig. 7a, *p < 0.05) and cardiotoxicity indicator (LDH) (Fig. 7b, *p < 0.05) of mice in the PTX and group were significantly higher than those in other groups. Additionally, various indicators in 4WJ-EGFR_apt_-miR-375-PTX group were improved compared with the 4WJ-miR-375-PTX group (Fig. 7a–c), indicating that EGFR_apt_ modification can reduce the toxicity and side effects of nano-drugs.

## Conclusions

In this study, a novel 4WJ-based RNA nanoparticle which was co-loaded with PTX and ESCC-suppressive miR-375 selected by RNA sequencing and cytological function verification and decorated with EGFR_apt_ (4WJ-EGFR_apt_-miR-375-PTX) was developed, and its anti-ESCC capacity was investigated. 4WJ-EGFR_apt_-miR-375-PTX possessed optimal thermal and pH stability. Moreover, PTX release was directly related to the esterase hydrolysis. In vitro analysis demonstrated that 4WJ-EGFR_apt_-miR-375-PTX could be internalized by KYSE-150 cells and penetrates into tumor spheroids with high efficiency, and thus, suppresses the proliferation of KYSE-150 cells more efficiently. More importantly, 4WJ-EGFR_apt_-miR-375-PTX was selectively accumulated in the tumor site via EPR effect and EGFR_apt_-mediated active targeting. The optimal therapeutic efficacy of 4WJ-EGFR_apt_-miR-375-PTX against KYSE-150-derived tumors, which was mediated by the enhanced PTX distribution in tumors and synergistic effect of miR-375 and PTX, was observed in vivo. Therefore, this delivery system offers a new targeted therapy strategy for the treatment of ESCC, with attractive prospects for its clinical application in transformation.

## Methods

### Materials

miRNA mimics including miR-375, miR-4776-5p, miR-1973, miR-1246, miR-139 were provided by Sangon Biotech. Paclitaxel (PTX) was purchased from Aladdin Bio-Chem Technology Co., LTD. Matrigel was ordered from BD Biosciences (San Jose, CA, USA) Annexin V/PI kit were bought from Dojindo Molecular Technologies, Inc. D-luciferin was ordered from Sigma-Aldrich (St. Louis, MO, USA). Anti-EGFR antibody was purchased from RriGene Technologies, Inc. Anti-Bax (Cat. No. 5023), Bcl-2 (Cat. No. 4223), Caspase-3 (Cat. No. 14220), Cyclin A2 (Cat. No. 4656), Cyclin B1 (Cat. No. 4135), Cyclin D1 (Cat. No. 55506) and E-Cadherin (Cat. No. 14472) antibodies were purchased from Cell Signaling Technology (Boston, MA, USA).

### Cell culture

Human esophageal cancer cell KYSE-150 was kept in our laboratory and identified by the China Center for Type Culture Collection (CCTCC). Luciferase-expressing KYSE-150 cells (Luc-KYSE-150) were constructed in our lab. Cells were maintained in DMEM containing both FBS (10%) and antibiotics (100 µL/ml streptomycin and 100 µL/ml penicillin) at 37 °C in humidified air environment containing 5% CO_2_.

### Mice

4–6-week-old BALB/c nude mice were purchased from Hangzhou Ziyuan Experimental Animal Technology Co., Ltd. (Hangzhou, China) and all experiments were approved by the Animal Care and Use Committee of The Affiliated Huaian No.1 People’s Hospital of Nanjing Medical University (DW-P-2021-001-01).

### Colony formation assay

To validate the function of miRNA mimics on KYSE-150 cells, KYSE-150 cells (400/mL) were cultured in 6-well plates and transfected with miRNA mimics (miR-375, miR-4776-5p, miR-1973, miR-1246, miR-139) by lipofectamine 3000. After 14 days culturing, cell colonies were fixed with 4% PFA, stained with a 0.1% crystal violet dye, photographed, and counted.

### Apoptosis analysis

2 × 10^5^ KYSE-150 cells that were respectively transfected with miRNA mimics (miR-375, miR-4776-5p, miR-1973, miR-1246, miR-139) were cultured in a 6-well plate for 48 h at 37 °C. The apoptotic cells were determined by Annexin V-FITC/PI staining and analyzed by flow cytometry (BD Accuri C6 Plus, Germany).

### Migration and invasion assays

Migration and invasion of KYSE-150 cells were detected using transwell chambers. For migration, KYSE-150 cells (4 × 10^4^/well) were cultured in 6-well plate for 24 h, and transfected with miRNA mimics (miR-375, miR-4776-5p, miR-1973, miR-1246, miR-139) by lipofectamine 3000. After transfection for 6 h, cells were collected and resuspended with serum-free DMEM. Cells were then seeded (2 × 10^4^) in the upper chamber of each insert and the medium with 10% FBS was placed in the lower chamber. After incubation for 48 h, cells on the upper layer of the membrane were discarded, the cells on the lower surface were fixed with methanal and stained with 0.1% crystal violet dye. The stained cells were then photographed and counted in three randomly selected fields. The invasion assays were performed using Matrigel-coated Transwell inserts with the same procedure as migration assay.

### EGFR expression in ESCC tissue and KYSE-150 cells

Tumor and adjacent esophageal tissues were obtained from 140 patients who underwent surgery for ESCC at the Department of Thoracic Surgery, the Affiliated Huai’an No.1 People’s Hospital of Nanjing Medical University. This study was approved by the ethics committee of Affiliated Huai’an No.1 People’s Hospital, Nanjing Medical University (YX-P-2020-055-01).

ESCC tissue microarrays (TMA) were prepared by Servicebio (Wuhan, China). After deparaffinization and rehydration, sections were treated for antigen retrieval for 5 min and blocked with 5% BSA for 1 h at room temperature. After 3 times washing, tissue sections were incubated with anti-EGFR antibody (1:150) for 3 h, and the EGFR expression was detected by the EnVision FLEX/HRP (Dako Denmark A/S). The intensity and extent of EGFR expression was finally determined and quantified using the histochemical scoring system (H-score).

To test the EGFR expression in EYSE-150 cells, cells (2 × 10^4^/well) were cultured on coverslips in 24-well plate for 24 h. After 3 times washing, cells were fixed with 4% paraformaldehyde for 20 min and blocked with 5% BSA for 30 min. After 3 times washing, cells were successively incubated with anti-EGFR antibody and Alexa 488 labeled goat anti-rabbit IgG. The expression of EGFR was finally observed by a confocal microscope (NIKON A1 +).

### Synthesis of RNA oligomers and PTX-N3

RNA oligomers were obtained from ExonanoRNA Biomedicine (Foshan, China), rG, rG, rC, rU, 2’F rC and 2’F rU phosphoramidites were purchased from Huaren Science and Technology Co., Ltd. (Wuhu, China). 2’O-propargyl rC and rU were ordered from Chemgene. RNA oligomers were purified by desalting using Glen Pak purification cartridges and gel electrophoresis (Bio-Rad). PTX-N3 prodrug was synthesized according to previous report. In brief, paclitaxel, N,N′-dicyclohexyl-carbodiimide, 4-(dimethylamino) pyridine and 6-azido-hexanoic acid were mixed and reacted in 10 mL dichloromethane with an equivalent ratio of 1:2:1:2. The reaction was carried out at 25 °C with stirring for 16 h. The crude product was yielded by filtration and rotary evaporation, and finally purified by silica gel chromatography.

### Construction of 4WJ-EGFR_apt_-miR-375-PTX nanoparticles

The nanoparticles including 4WJ, 4WJ-EGFR_apt_, 4WJ-miR-375, 4WJ-EGFR_apt_-miR-375, 4WJ-PTX, 4WJ-EGFR_apt_-PTX, 4WJ-miR-375-PTX and 4WJ-EGFR_apt_-miR-375-PTX were constructed and provided by ExonanoRNA LLC.

For synthesis of RNA oligomer-PTX, RNA-6 alkynes oligomers (six 2-propargyl nucleotides in each oligomer) were mixed in DMSO, Copper sulfate/Tris[(1-benzyl-1H-1,2,3-triazol-4-yl)methyl]amine (TBTA) and sodium ascorbate were added and vibrated at 4 °C for 16 h. After reaction, the RNA oligomers-PTX was precipitated with 3 M sodium acetate and 100% ethanal. The compound was confirmed and purified by 16% (w/v) native PAGE in TBE buffer (89 mM Tris base, 200 mM boric acid and 2 mM EDTA).

For the nanoparticles assembling, four RNA-6PTXs oligomers (4WJA, 4WJB or 4WJB-miR-375, 4WJC or 4WJC-miR-375, 4WJD) were mixed in TES buffer (50 mM Tris pH = 8.0, 50 mM NaCl, 1 mM EDTA) at equimolar concentrations, denatured at 90 °C for 10 min and cooled to 4 °C gradually. The nanoparticles including 4WJ, 4WJ-EGFR_apt_, 4WJ-miR-375, 4WJ-EGFR_apt_-miR-375, 4WJ-PTX, 4WJ-EGFR_apt_-PTX, 4WJ-miR-375-PTX and 4WJ-EGFR_apt_-miR-375-PTX were identified by 12% (w/v) native PAGE in TBE buffer. The sequences of RNA-6PTXs, miR-375 and EGFR aptamer are (lower cases indicate 2′-F nucleotides, ^ indicate the points for PTX conjugation and the underlined letters indicate EGFR aptamer):

**4WJA**: 5′^-uuA GG^u AAA G^cc Acc uGc AGG uGc uAc ^cGA uG^u AAu u^cA A -3′; **4WJB**: 5'^-uuG AA^u uAc A^uc GGu AGc AcG GGc uGu G^cG AGG ^cuG AA^c AG -3'; **4WJB-miR-375**: 5'^-uuG AA^u uAc A^uc GGu AGc AcG GGc uGu G^cG AGG ^cuG AA^c AG GcG AcG AGc ccc UcG cAc AAA cc-3'; **4WJC**: 5′^-cuG uu^c AGc c^uc GcA cAG ccA GcA ^cGc Ac^c uGA A^uA GGu -3’; **4WJC-EGFR**_**apt**_: 5′^-cuG uu^c AGc c^uc GcA cAG ccA GcA ^cGc Ac^c uGA A^uA GGu Gcc uuA GuA AcG uGc uuu GAu Guc GAu ucG AcA GGA GGc -3’; **4WJD**: 5’^- ccu Au^u cAG G^uG cGu Gcu GGG cuG cAG G^uG Gcu u^uA cc^u AA -3′; **miR-375**: 5'- UUU GUU CGU UCG GCU CGC GUG A -3'.

### DLS measurement

To detect size distribution and surface zeta potential of 4WJ, 4WJ-EGFR_apt_, 4WJ-miR-375, 4WJ-EGFR_apt_-miR-375, 4WJ-PTX, 4WJ-EGFR_apt_-PTX, 4WJ-miR-375-PTX and 4WJ-EGFR_apt_-miR-375-PTX, samples were dissolved in RNase free ddH_2_O and detected by dynamic light scattering (PSS Z3000).

### Atomic force microscopy

10 μL of nanoparticles (4WJ, 4WJ-EGFR_apt_, 4WJ-miR-375, 4WJ-EGFR_apt_-miR-375, 4WJ-PTX, 4WJ-EGFR_apt_-PTX, 4WJ-miR-375-PTX and 4WJ-EGFR_apt_-miR-375-PTX) were deposited on freshly cleaved mica and dried overnight at room temperature. Then the mica surface was scanned by an atomic force microscope (DMFASTSCAN2-SYS, Bruker).

### Ultraviolet–visible (UV–Vis) spectroscopy

Ultraviolet–Visible (UV–Vis) Spectroscopy was determined by NANODROP 2000 (Thermo SCIENTIFIC) in transmission mode at a wavelength range of 200–800 nm.

### Tm analysis by RT-PCR

RNA nanoparticles including 4WJ, 4WJ-EGFRapt, 4WJ-miR-375, 4WJ-EGFR_apt_-miR-375, 4WJ-PTX, 4WJ-EGFRapt-PTX, 4WJ-miR-375-PTX and 4WJ-EGFRapt-miR-375-PTX were respectively mixed with SYBR Green II and added to 96-well plate. Samples were denatured at 95 °C for 5 min and annealed to 20 °C at a rate of 0.11 °C /s. The SYBR Green II signals were then examined by LightCycler®480 RT-PCR. Tm value was calculated by three independent measurements.

### Enzymatic stability assay

5 μM of 4WJ-EGFR_apt_-miR-375-PTX was incubated with RNase (0.1 μg/μL) at 37 °C for different times (0, 1, 3, 6, 12 and 24 h). The samples were then examined by 2% agarose gel electrophoresis and the percentage of intact nanoparticles (intensity of the band at a time point/intensity of the band at 0 h) was quantified by Image J.

### pH stability analysis

4WJ-EGFR_apt_-miR-375-PTX was respectively incubated in PBS with pH4, 5.5 and 7.4 for different times (0, 0.5, 1, 4, 8, 12, 24, 36 and 48 h). The resulting samples were examined by 16% native acrylamide PAGE and the percentage of intact nanoparticles was quantified by Image J.

### PTX release profile

4WJ-EGFRapt-miR-375-PTX was incubated in PBS with 50% FBS for 0, 1, 2, 8, 12, 24, 36 and 48 h. The 16% native acrylamide PAGE electrophoresis was carried out to test and quantify the drug release rate.

### Confocal microscopy imaging

To investigate the cellular binding and uptake efficiency, KYSE-150 cells were cultured in chamber slides (2 × 10^4^/well) for 24 h. Then, AF647-labeled RNA nanoparticles (400 nM) including 4WJ, 4WJ-EGFR_apt_, 4WJ-miR-375-PTX and 4WJ-EGFR_apt_-miR-375-PTX were respectively added and incubated at 37 °C for 12 and 24 h. Cells were then washed 3 times with PBS, fixed with 4% PFA for 10 min at room temperature followed by staining with DAPI. The AF647 signals in cells was examined and quantified using a confocal microscope (NIKON A1 +).

To study the penetration capacity of nanoparticles, 3D multicellular tumor spheroids were prepared by suspending KYSE-150 cells (1 × 10^3^) with DMEM/Matrigel (1:1, v/v) in 35 mm culture dishes. After 10–14 days culturing, culture medium was removed and cells were washed with PBS for 3 times, then the AF647-labeled 4WJ-miR-375-PTX and 4WJ-EGFR_apt_-miR-375-PTX (800 nM) were respectively added and incubated with tumor spheroids for 12 and 24 h. Then the spheroids were fixed with 4% PFA, stained with DAPI and the AF647 signals were detected and quantified by confocal microscope.

### In vitro cytotoxicity assay

KYSE-150 cells were seeded in cell culture E-plate at density of 5 × 10^3^/well and incubated overnight at 37 °C. Cells were then respectively treated with PBS, 4WJ, 4WJ-EGFR_apt_, 4WJ-miR-375, 4WJ-EGFR_apt_-miR-375, PTX, 4WJ-PTX, 4WJ-EGFR_apt_-PTX, 4WJ-miR-375-PTX and 4WJ-EGFR_apt_-miR-375-PTX for 48 h. The cell growth curves were automatically recorded on the xCELLigence System (Roche Applied Sciences) in real-time.

Growth of 3D tumor spheroids was further tested by treatment with aforementioned nanoparticles and PTX every 5 days for 2 times. Then the spheroids were imaged and the size was calculated.

### Western blot

KYSE-150 cells (4 × 10^5^) were cultured in 6-well plates and treated with 10 nM of nanoparticles including 4WJ, 4WJ-EGFR_apt_, 4WJ-miR-375, 4WJ-EGFR_apt_-miR-375, 4WJ-PTX, 4WJ-EGFR_apt_-PTX, 4WJ-miR-375-PTX, 4WJ-EGFR_apt_-miR-375-PTX and 240 nM of PTX for 48 h, then the cells were lysed and subjected to SDS/PAGE.

After transferring the proteins onto polyvinylidene fluoride membrane, the membrane was blocked and incubated with antibodies against Bax (1:2000), Bcl2 (1:2000), caspase-3 (1:2000), Cyclin A2 (1:4000), Cyclin B1 (1:4000), Cyclin D1 (1:2000), and E-cadherin (1:2000) overnight at 4 °C. After three times washing, membrane was incubated with secondary antibody for 1 h. Finally, the proteins were visualized using an enhanced chemiluminescence (ECL) detection reagent (Tanon).

### Biodistribution of nanoparticles

To verify whether EGFR_apt_ could enhance the distribution of nanoparticles in KYSE-150-derived tumor tissues, 5 nmol of AF647 labeled 4WJ, 4WJ-EGFR_apt_, 4WJ-miR-375-PTX and 4WJ-EGFR_apt_-miR-375-PTX were intravenously injected into KYSE-150-bearing BALB/c nude mice, live imaging was performed at 2, 4, 6 and 8 h after administration. Mice were sacrificed, the organs including livers, lungs, kidneys, spleens, hearts, and tumor tissues were collected, the distribution of nanoparticles was scanned and quantified by live imaging system (Bruker FX Pro).

### Xenograft tumor model

Female BALB/c nude mice (6 − 8 weeks) were subcutaneously injected with KYSE-150 cells (1 × 10^7^/mouse). When tumors reached approximately 50 mm^3^ in volume, the mice were then randomly divided to different groups for following studies.

### In vivo tumor suppression evaluation

KYSE-150 tumor-bearing mice were randomly assigned to 10 groups and intravenously treated with PBS, 4WJ (5 nmol), 4WJ-EGFR_apt_ (5 nmol), 4WJ-miR-375 (5 nmol), 4WJ-EGFR_apt_-miR-375 (5 nmol), PTX (120 nmol), 4WJ-PTX (5 nmol 4WJ with 120 nmol PTX), 4WJ-EGFR_apt_-PTX, 4WJ-miR-375-PTX and 4WJ-EGFR_apt_-miR-375-PTX every 7 days for 5 times. Tumor size and mice weight were measured every 3 days, the luciferase signals in tumors were detected every 7 days. Mice were sacrificed 7 days after last administration, the peripheral blood was collected, the blood routine examination was carried out and the biomarkers for the liver (ALT, AST, and ALB), heart (LDH, CK-MB, and CK), kidney (BUN, CREA), glucose and lipid level in serum were tested by an animal biochemical analyzer.

Livers, lungs, hearts, kidneys, spleens, and tumors were removed, tumors were photographed, HE staining was performed to detect the pathological changes, IHC staining of Ki67 was carried out to analyze the proliferation of cancer cells.

### Ki67 staining

Paraffin-embedded, 5 μm thick tumor tissues were dried at 60 °C for 2 h, deparaffinized in xylene and hydrated in decreasing alcohol series (100%, 95%, 85%, 70%) before Ki67 staining. Antigen retrieval was carried out by boiling the slides for 5 min. After 3-time washes with PBS, endogenous peroxidase was inactivated with 0.3% hydrogen peroxide at room temperature for 15 min. After 3-time washes, slides were blocked with 5% BSA for 1 h and then stained with Ki67 for 3 h at room temperature.

The sections were washed and incubated with HRP-labeled goat anti-rabbit IgG at room temperature for 30 min. After coloring with DAB, the counterstaining, dehydration, and vitrification were successively performed. Finally, the slides were mounted and the percentage of Ki67 positive cells was calculated.

### Statistical analysis

All data are presented as mean ± standard deviation and analyzed by GraphPad Prism software (version 8.0). One- and two-way analysis of variance were used for multiple group comparisons. The unpaired t-test was used to compare the differences between two groups. *p < 0.05 was considered to be statistically significant.

## Supplementary Information

**Additional file 1**: **Figure S1**. miRNAs detection in ESCC tissues. a) RNA sequencing was performed to detect the miRNAs expression in ESCC and corresponding adjacent tissues. b) Major differential expression of miRNAs.** Figure S2**. Expression of EGFR in esophageal carcinoma tissues identified by TCGA database. **p<0.01. **Figure S3. **Validation of the EGFR expression in ESCC tissues. a) Expression of EGFR in ESCC tumor and adjacent tissues by IHC. b) Average staning intensity of EGFR in 140 ESCC tumor and corresponding adjacent tissues. ****p<0.0001. **Figure S4. **Expression of EGFR in KYSE-150 cells. Scale bar: 50μm. **Figure S5**. Construction of Alexa Fluor 647 labeled 4WJ. Synthesis of Alexa Fluor 647 labeled 4WJ by four RNA oligomers (4WJA, 4WJB-EGFR_apt_, 4WJC and 4WJD-AF647). **Figure S6**. Uptake efficiency of nanoparticles by KYSE-150 cells. a) AF647 labeled 4WJ and 4WJ-EGFR_apt_ were incubated with KYSE-150 cells for 24 h, the AF647 signals in cells was observed by confocal imaging. b) AF647 positive KYSE-150 cells were quantified by flow cytometry. Scale bar: 25 μm. **Figure S7.** Biodistribution of nanoparticles in ESCC tumor mice. a) AF647 labeled 4WJ and 4WJ-EGFR_apt_ were intravenously injected into ESCC tumor mice, the live imaging was performed after administration for 8 h. b) Mice were sacrificed after injection for 8 h, the distribution of 4WJ and 4WJ-EGFR_apt_ in major organs including livers, lungs, kidneys, spleens, hearts and tumors was analyzed by live imaging. **Figure S8. **Synthesis of PTX-N3 and identification by HPLC. **Figure S9. **Synthesis and verification of RNA oligomer-PTX. RNA oligomers-PTX (4WJA-6PTX, 4WJB-6PTX, 4WJC-6PTX, 4WJD-6PTX, 4WJC-EGFR_apt_-6PTX and 4WJB-miR-375-6PTX) were synthesized, precipitated, and purified by 16% native PAGE electrophoresis. **Figure S10.** Representative atomic force microscopy image of 4WJ, 4WJ-EGFR_apt_, 4WJ-miR-375, 4WJ-EGFR_apt_-miR-375, 4WJ-PTX, 4WJ-EGFR_apt_-PTX and 4WJ-miR-375-PTX. **Figure S11.** Tm curves and Tm values of nanoparticles including 4WJ, 4WJ-EGFR_apt_, 4WJ-miR-375, 4WJ-EGFR_apt_-miR-375, 4WJ-PTX, 4WJ-EGFR_apt_-PTX and 4WJ-miR-375-PTX. **Figure S12.** Proliferation of KYSE-150 cells after treatment with PTX and nanodrugs. KYSE-150 cells were cultured in E-plate and incubated with PTX, 4WJ-PTX, 4WJ-EGFR_apt_-PTX, 4WJ-miR-375-PTX and 4WJ-EGFR_apt_-miR-375-PTX in different concentrations, the proliferation curves were recorded by the xCELLigence system in real-time. **Figure S13.** Ki67 expression in tumor tissues. KYSE-150 tumor-bearing mice were treated with PBS, 4WJ, 4WJ-EGFR_apt_, 4WJ-miR-375, 4WJ-EGFR_apt_-miR-375, PTX, 4WJ-PTX, 4WJ-EGFR_apt_-PTX, 4WJ-miR-375-PTX and 4WJ-EGFR_apt_-miR-375-PTX for 5 times, then Ki-67-positive cells (brown) in tumors were detected by immunohistochemistry. Scale bar: 100 μm. **Figure S14. **Mice body weight changes. Body weight of KYSE-150 tumor-bearing mice was measured every 3 days. **Figure S15.** Pathological changes of hearts, livers, spleens, lungs and kidneys was analyzed by HE staining**. **KYSE-150 tumor-bearing mice were treated with PBS, 4WJ, 4WJ-EGFR_apt_, 4WJ-miR-375, 4WJ-EGFR_apt_-miR-375, PTX, 4WJ-PTX, 4WJ-EGFR_apt_-PTX, 4WJ-miR-375-PTX and 4WJ-EGFR_apt_-miR-375-PTX for 5 times, then the major organs were collected and the HE staning was performed to detect the pathological changes. Scale bar:100 μm.

## Data Availability

All data generated and analyzed in this study are included in this published article.

## Abbreviations

4WJ: Four-way junction
4WJ-EGFR_apt_: EGFR_apt_-modified 4WJ carrier
AFM: Atomic force microscopy
ALB: Albumin
ALT: Alanine transaminase
AST: Aspartate transaminase
BUN: Blood urea nitrogen
CCTCC: China Center for Type Culture Collection
CK: Creatine kinase
CK-MB: Creatine kinase myocardial band
CREA: Creatinine
DLS: Dynamic light scattering
EGFR: Epidermal growth factor receptor
EGFR_apt_: Aptamer modification of EGFR
EPR: Enhanced permeability and retention
ESCC: Esophageal squamous cell carcinoma
LDH: Lactate dehydrogenase
miRNA: MicroRNA
pRNA: Packaging RNA
PTX: Paclitaxel
SELEX: Systematic evolution of ligands by exponential enrichment
TBTA: Tris[(1-benzyl-1H-1,2,3-triazol-4-yl)methyl]amine
TMA: Tissue microarray
UV–Vis: Ultraviolet–Visible
