# Supplementary material for: Self-assembled RNA nanocarrier-mediated chemotherapy combined with molecular targeting in the treatment of esophageal squamous cell carcinoma
Source: J Nanobiotechnology. 2021 Nov 25;19:388. doi: 10.1186/s12951-021-01135-5 (PMC8614048; doi:10.1186/s12951-021-01135-5)
Supplement: Supplementary file 1 — Additional file 1: Figure S1. miRNAs detection in ESCC tissues. a) RNA sequencing was performed to detect the miRNAs expression in ESCC and corresponding adjacent tissues. b) Major differential expression of miRNAs. Figure S2. Expression of EGFR in esophageal carcinoma tissues identified by TCGA database. **p<0.01. Figure S3. Validation of the EGFR expression in ESCC tissues. a) Expression of EGFR in ESCC tumor and adjacent tissues by IHC. b) Average staning intensity of EGFR in 140 ESCC tumor and corresponding adjacent tissues. ****p<0.0001. Figure S4. Expression of EGFR in KYSE-150 cells. Scale bar: 50μm. Figure S5. Construction of Alexa Fluor 647 labeled 4WJ. Synthesis of Alexa Fluor 647 labeled 4WJ by four RNA oligomers (4WJA, 4WJB-EGFRapt, 4WJC and 4WJD-AF647). Figure S6. Uptake efficiency of nanoparticles by KYSE-150 cells. a) AF647 labeled 4WJ and 4WJ-EGFRapt were incubated with KYSE-150 cells for 24 h, the AF647 signals in cells was observed by confocal imaging. b) AF647 positive KYSE-150 cells were quantified by flow cytometry. Scale bar: 25 μm. Figure S7. Biodistribution of nanoparticles in ESCC tumor mice. a) AF647 labeled 4WJ and 4WJ-EGFRapt were intravenously injected into ESCC tumor mice, the live imaging was performed after administration for 8 h. b) Mice were sacrificed after injection for 8 h, the distribution of 4WJ and 4WJ-EGFRapt in major organs including livers, lungs, kidneys, spleens, hearts and tumors was analyzed by live imaging. Figure S8. Synthesis of PTX-N3 and identification by HPLC. Figure S9. Synthesis and verification of RNA oligomer-PTX. RNA oligomers-PTX (4WJA-6PTX, 4WJB-6PTX, 4WJC-6PTX, 4WJD-6PTX, 4WJC-EGFRapt-6PTX and 4WJB-miR-375-6PTX) were synthesized, precipitated, and purified by 16% native PAGE electrophoresis. Figure S10. Representative atomic force microscopy image of 4WJ, 4WJ-EGFRapt, 4WJ-miR-375, 4WJ-EGFRapt-miR-375, 4WJ-PTX, 4WJ-EGFRapt-PTX and 4WJ-miR-375-PTX. Figure S11. Tm curves and Tm values of nanoparticles includi [file 12951_2021_1135_MOESM1_ESM.docx]

Self -assembled RNA nanocarrier-mediated chemotherapy combined with molecular targeting in the treatment of esophageal squamous cell carcinoma


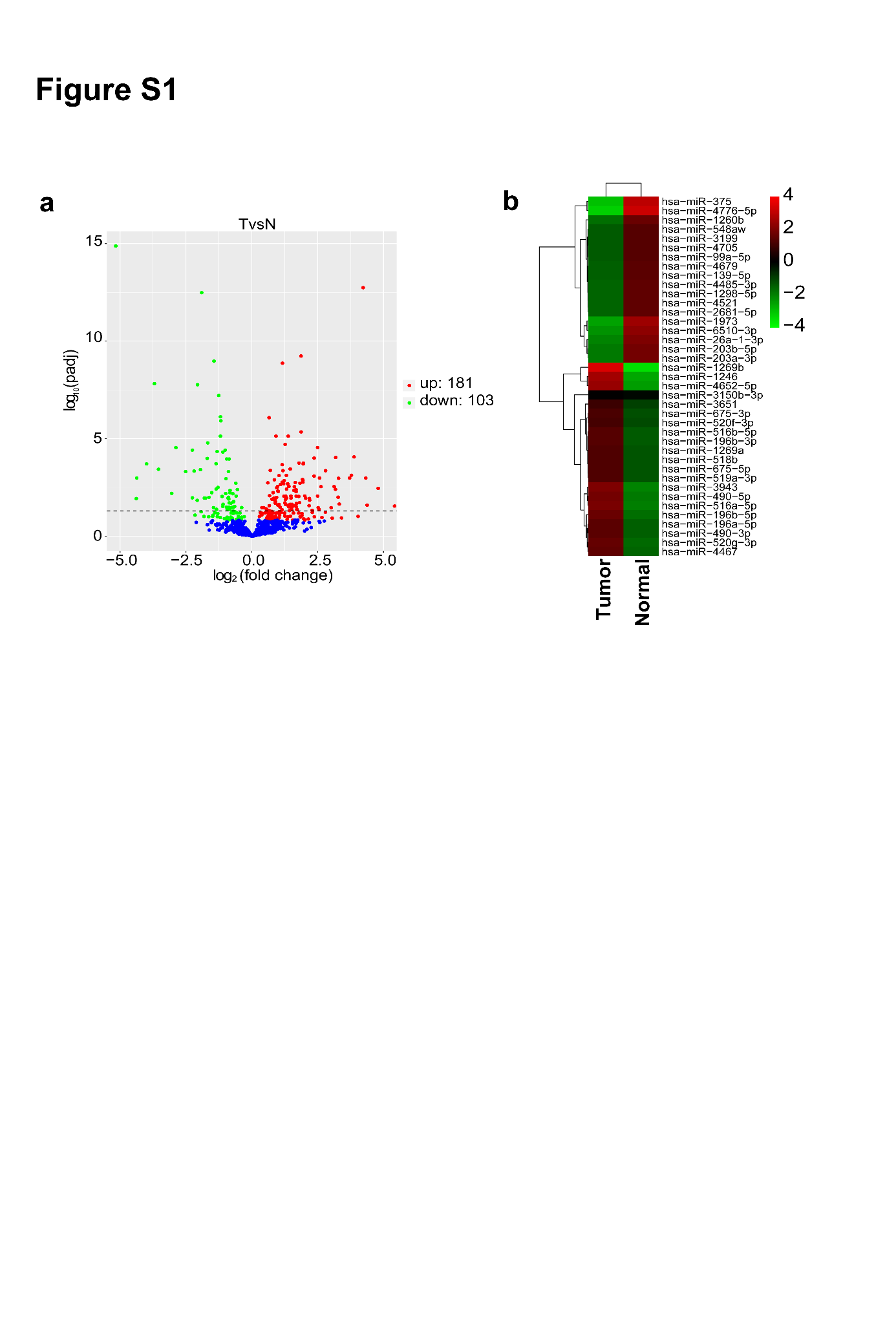


**Figure S1. miRNAs detection in ESCC tissues.** a) RNA sequencing was performed to detect the miRNAs expression in ESCC and corresponding adjacent tissues. b) Major differential expression of miRNAs.


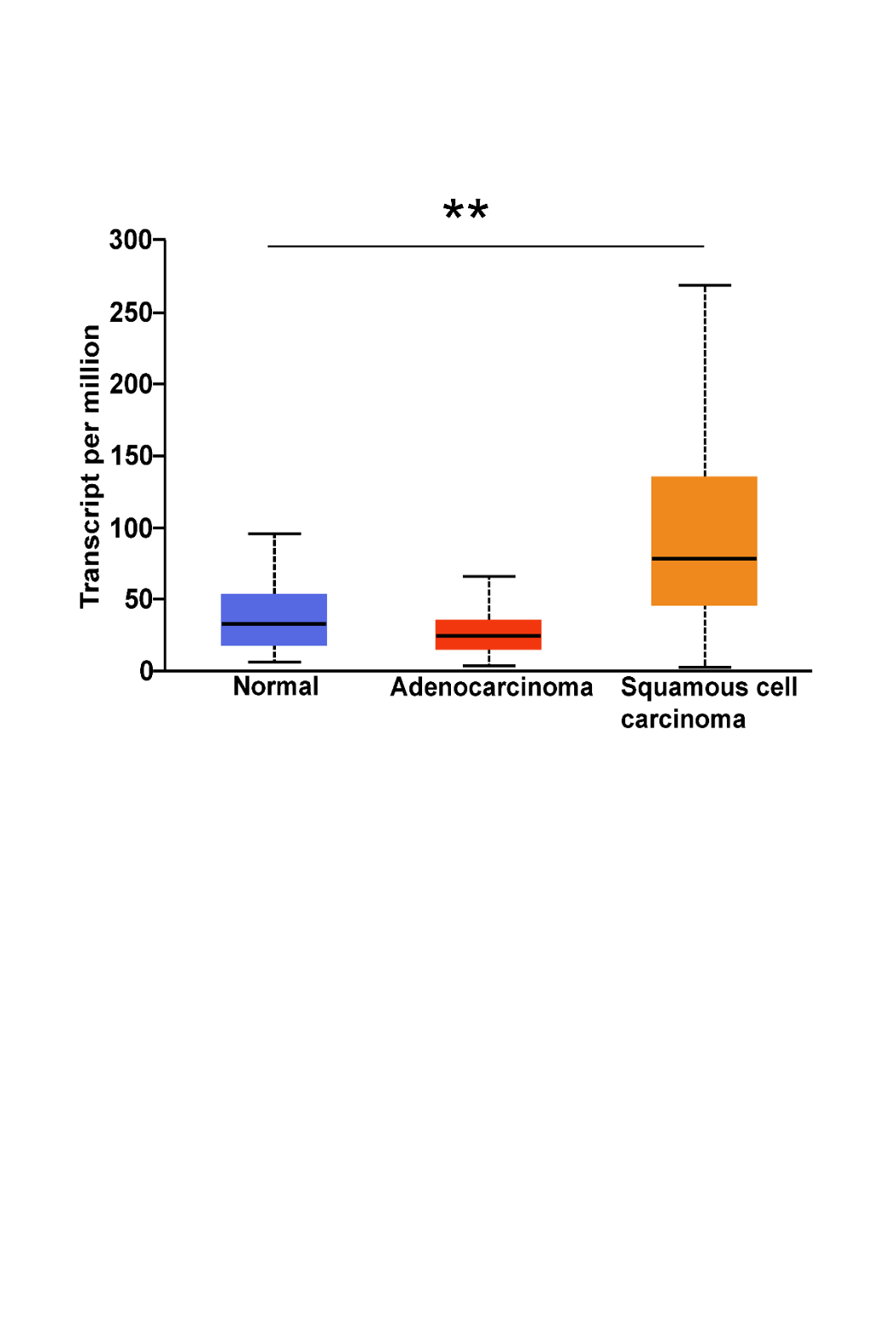


**Figure S2**. Expression of EGFR in esophageal carcinoma tissues identified by TCGA database. ***p*<0.01.


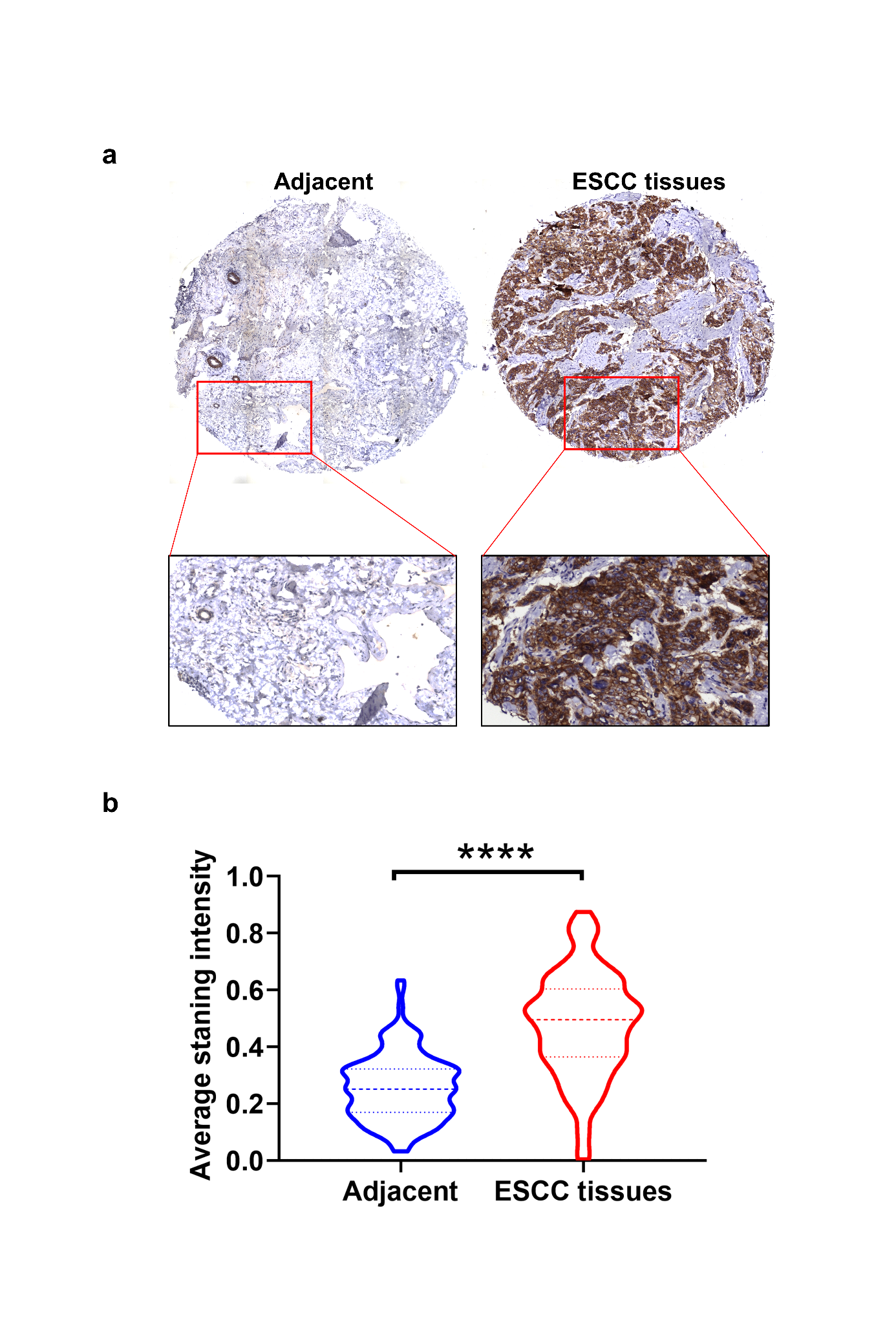


**Figure S3. Validation of the EGFR expression in ESCC tissues.** a) Expression of EGFR in ESCC tumor and adjacent tissues by IHC. b) Average staning intensity of EGFR in 140 ESCC tumor and corresponding adjacent tissues. *****p*<0.0001.


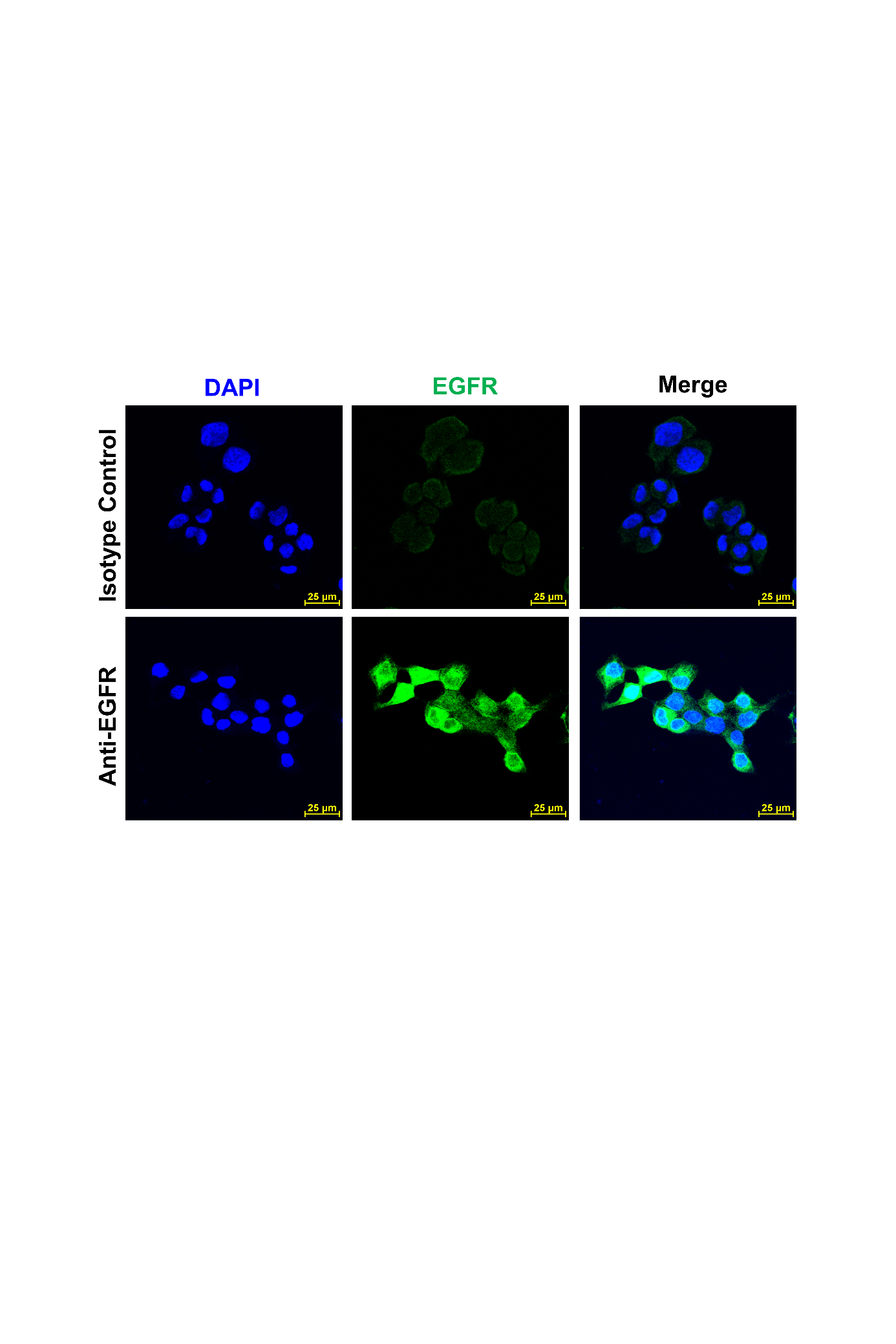


**Figure S4. Expression of EGFR in KYSE-150 cells.** Scale bar: 50μm.


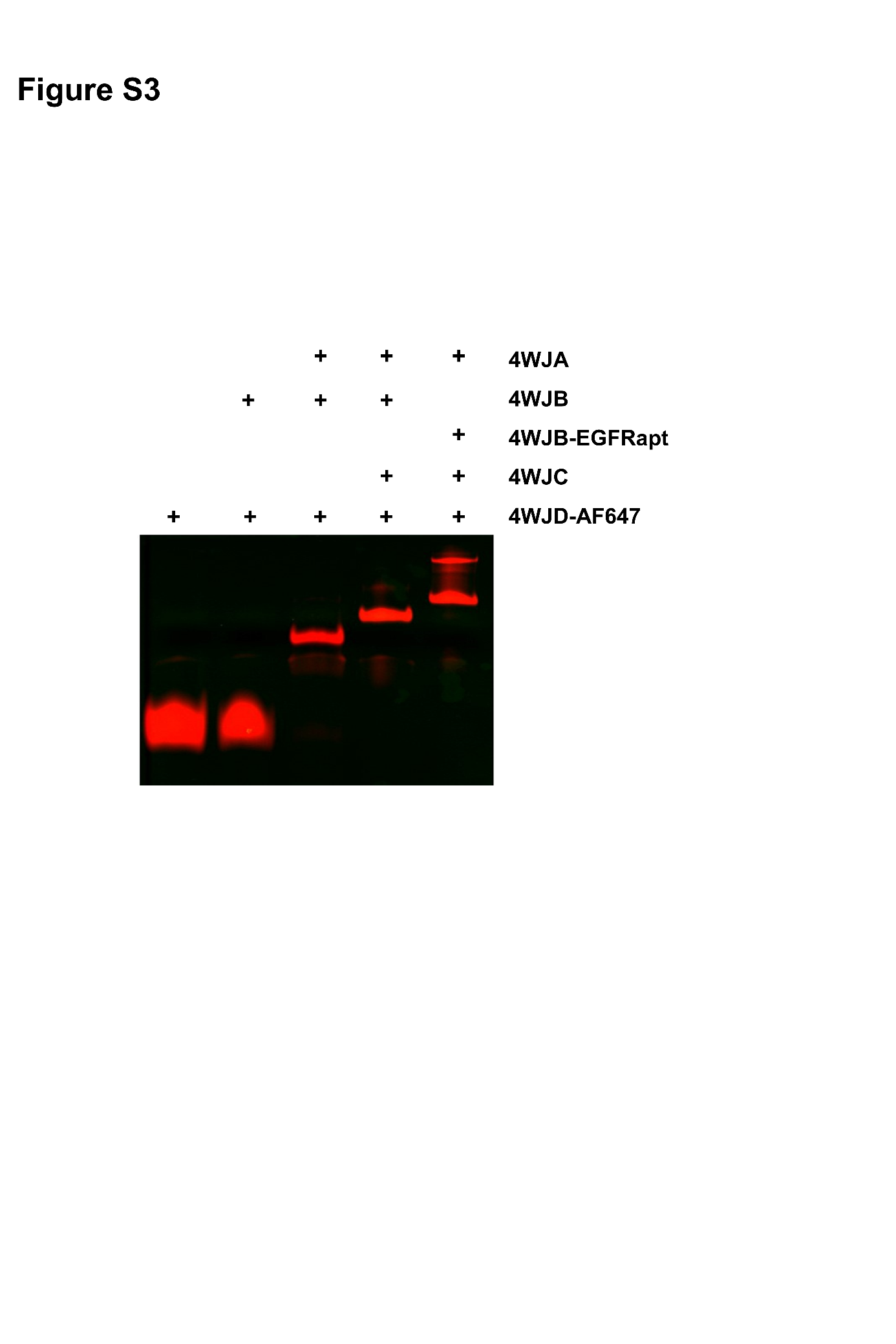


**Figure S5. Construction of Alexa Fluor 647 labeled 4WJ.** Synthesis of Alexa Fluor 647 labeled 4WJ by four RNA oligomers (4WJA, 4WJB-EGFR_apt_, 4WJC and 4WJD-AF647).


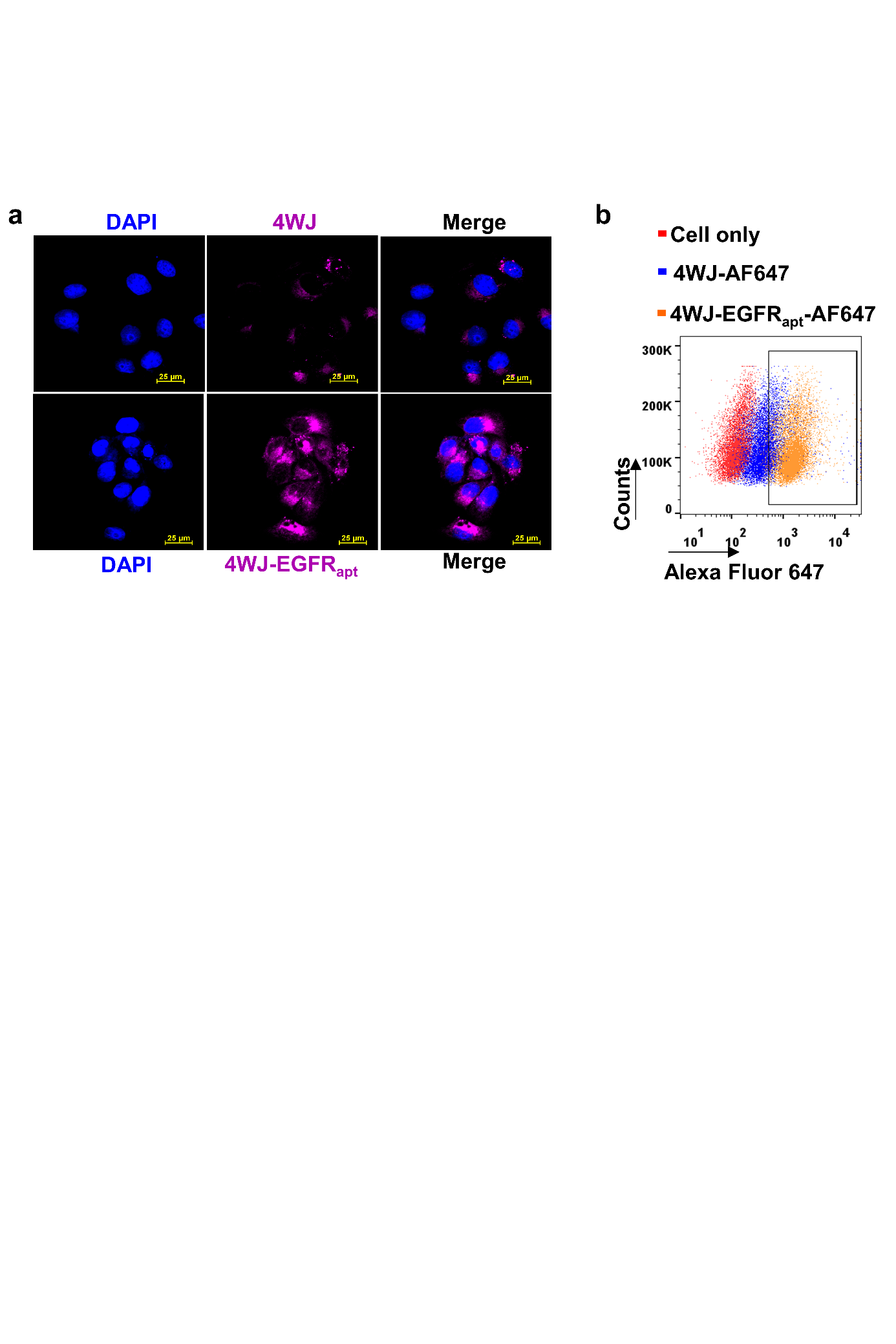


**Figure S6. Uptake efficiency of nanoparticles by KYSE-150 cells.** a) AF647 labeled 4WJ and 4WJ-EGFR_apt_ were incubated with KYSE-150 cells for 24 h, the AF647 signals in cells was observed by confocal imaging. b) AF647 positive KYSE-150 cells were quantified by flow cytometry. Scale bar: 25 μm.


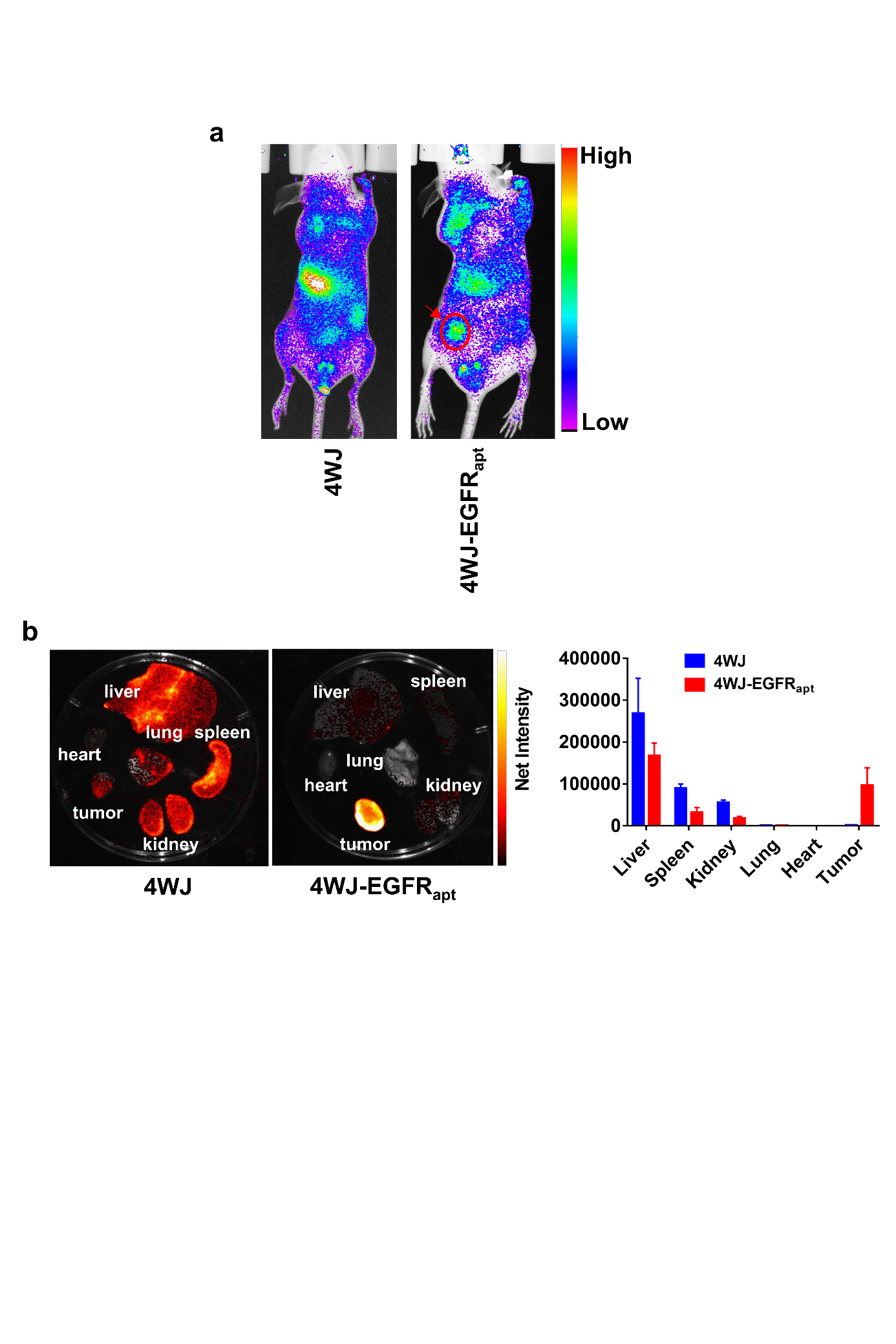


**Figure S7. Biodistribution of nanoparticles in ESCC tumor mice.** a) AF647 labeled 4WJ and 4WJ-EGFR_apt_ were intravenously injected into ESCC tumor mice, the live imaging was performed after administration for 8 h. b) Mice were sacrificed after injection for 8 h, the distribution of 4WJ and 4WJ-EGFR_apt_ in major organs including livers, lungs, kidneys, spleens, hearts and tumors was analyzed by live imaging.


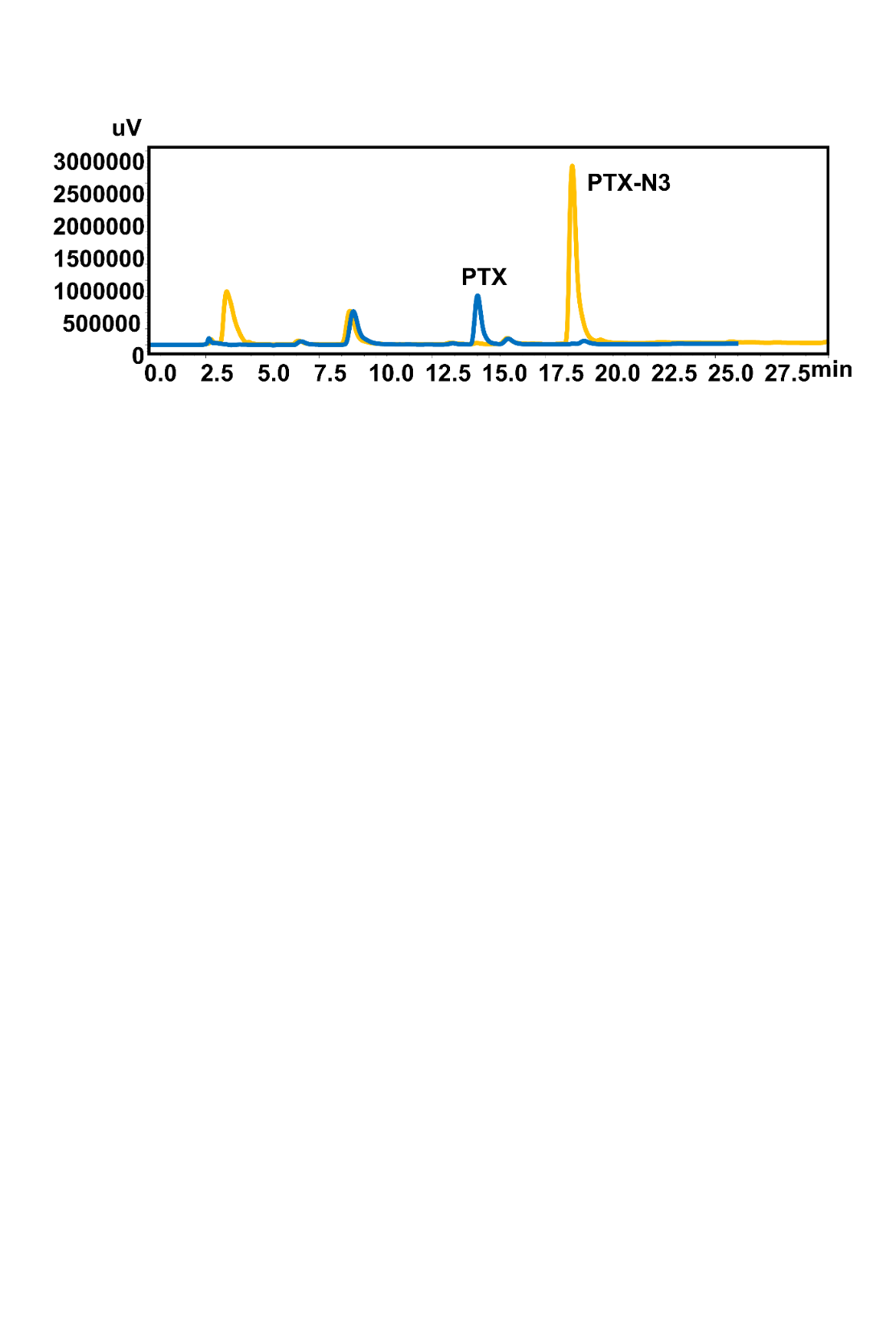


**Figure S8.** Synthesis of PTX-N3 and identification by HPLC.


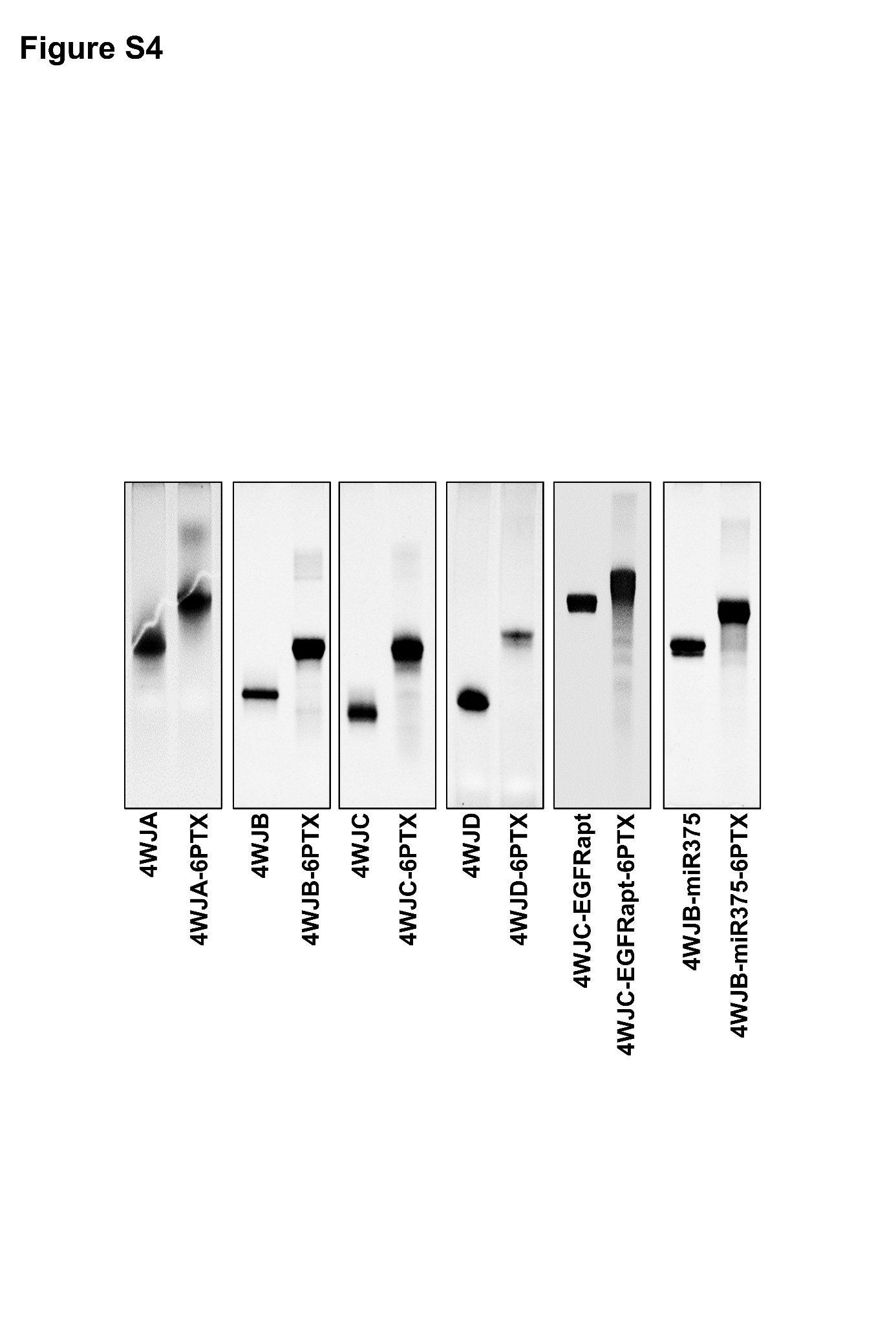
 **Figure S9. Synthesis and verification of RNA oligomer-PTX.** RNA oligomers-PTX (4WJA-6PTX, 4WJB-6PTX, 4WJC-6PTX, 4WJD-6PTX, 4WJC-EGFR_apt_-6PTX and 4WJB-miR-375-6PTX) were synthesized, precipitated, and purified by 16% native PAGE electrophoresis.


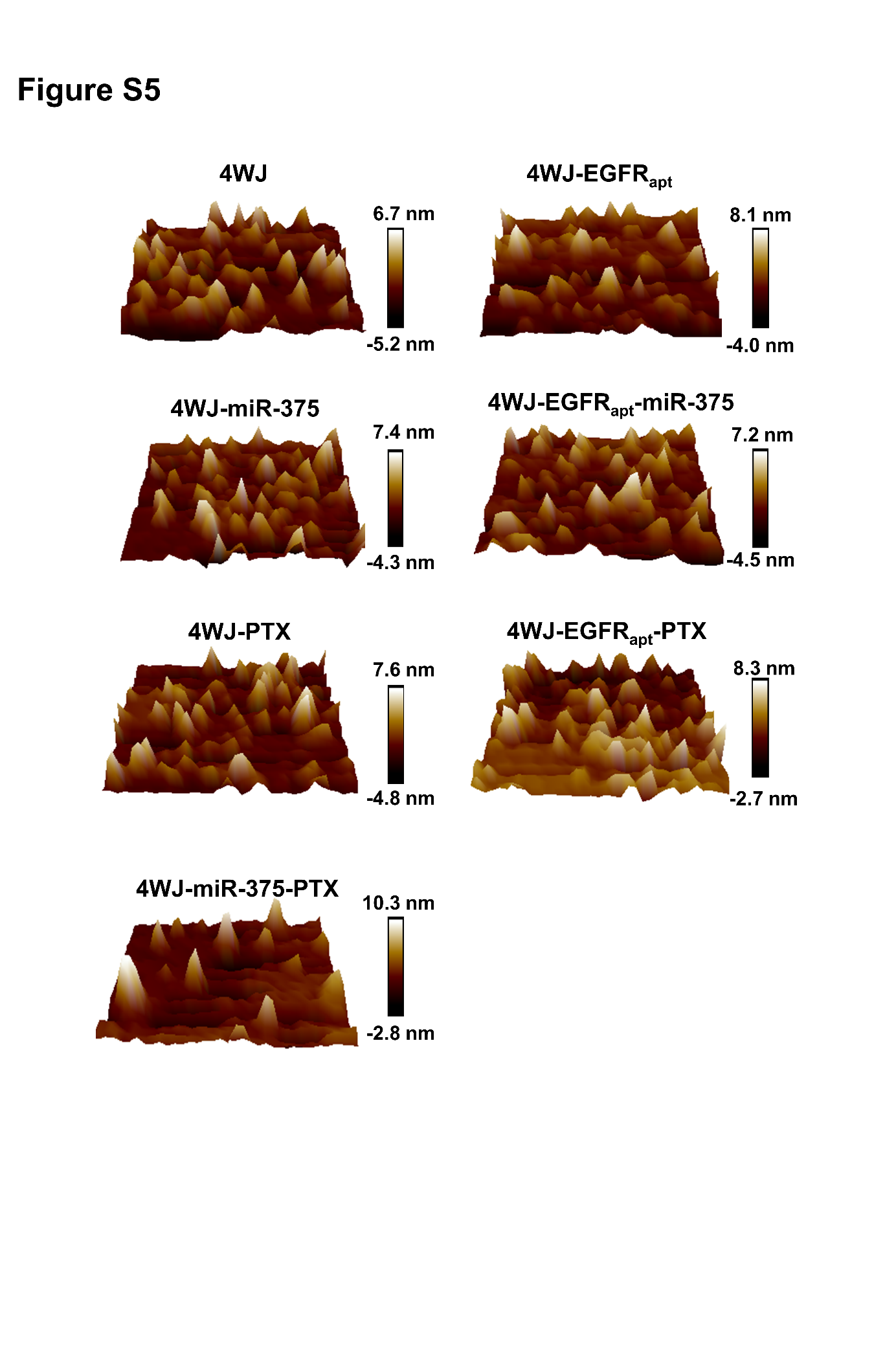


**Figure S10.** Representative atomic force microscopy image of 4WJ, 4WJ-EGFR_apt_, 4WJ-miR-375, 4WJ-EGFR_apt_-miR-375, 4WJ-PTX, 4WJ-EGFR_apt_-PTX and 4WJ-miR-375-PTX.


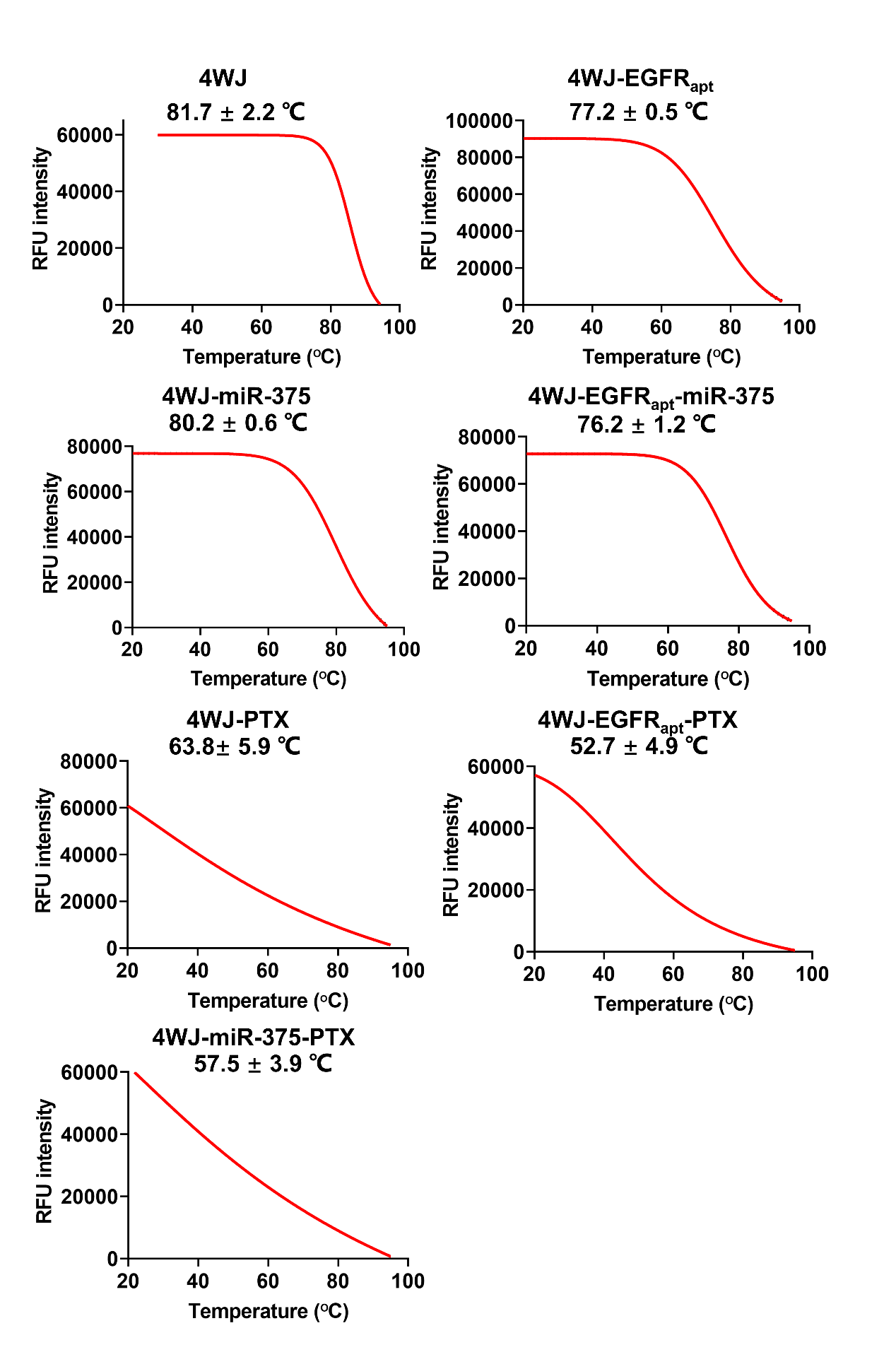


**Figure S11.** Tm curves and Tm values of nanoparticles including 4WJ, 4WJ-EGFR_apt_, 4WJ-miR-375, 4WJ-EGFR_apt_-miR-375, 4WJ-PTX, 4WJ-EGFR_apt_-PTX and 4WJ-miR-375-PTX.


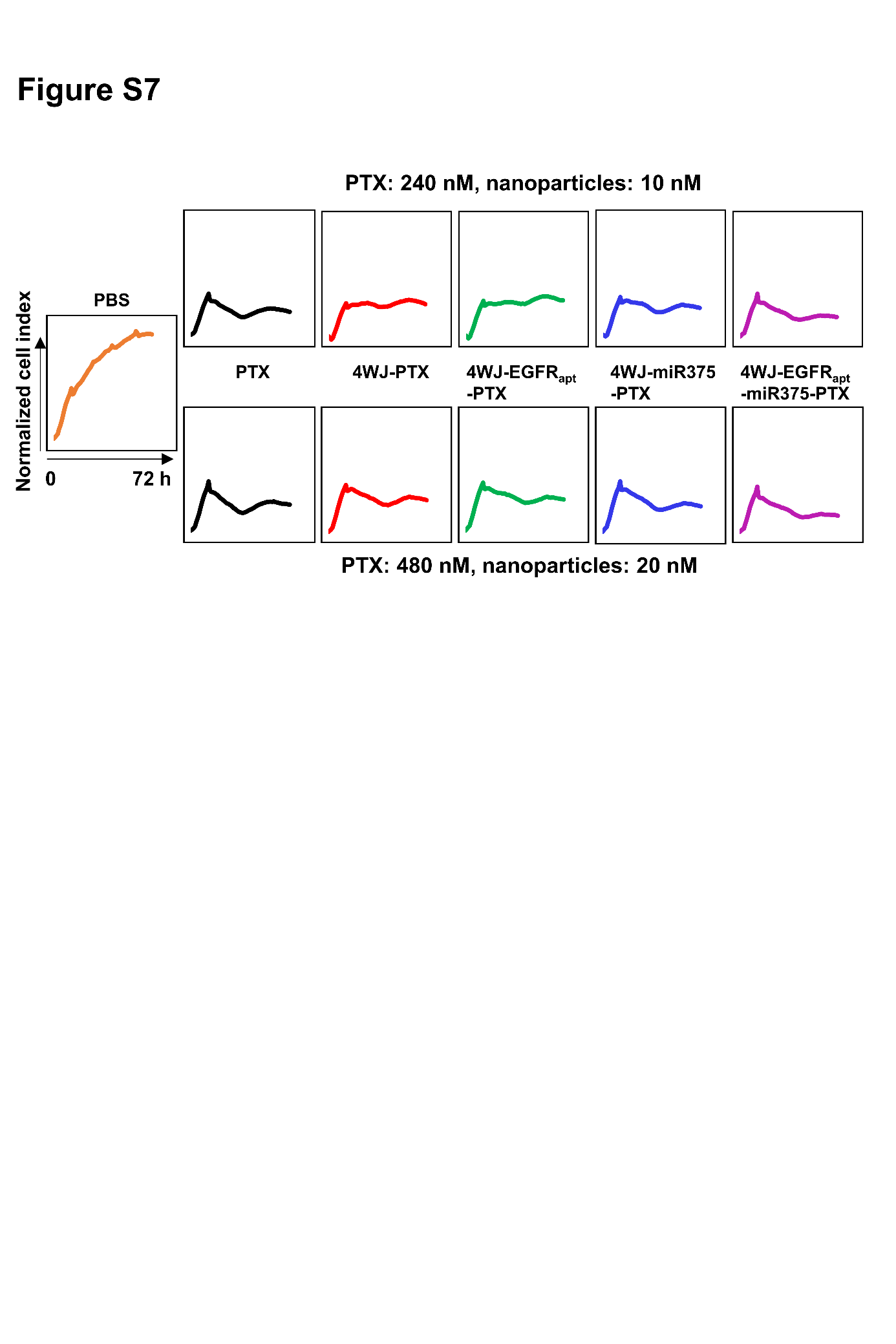


**Figure S12.** **Proliferation of KYSE-150 cells** **after treatment with PTX and nanodrugs.** KYSE-150 cells were cultured in E-plate and incubated with PTX, 4WJ-PTX, 4WJ-EGFR_apt_-PTX, 4WJ-miR-375-PTX and 4WJ-EGFR_apt_-miR-375-PTX in different concentrations, the proliferation curves were recorded by the xCELLigence system in real-time.


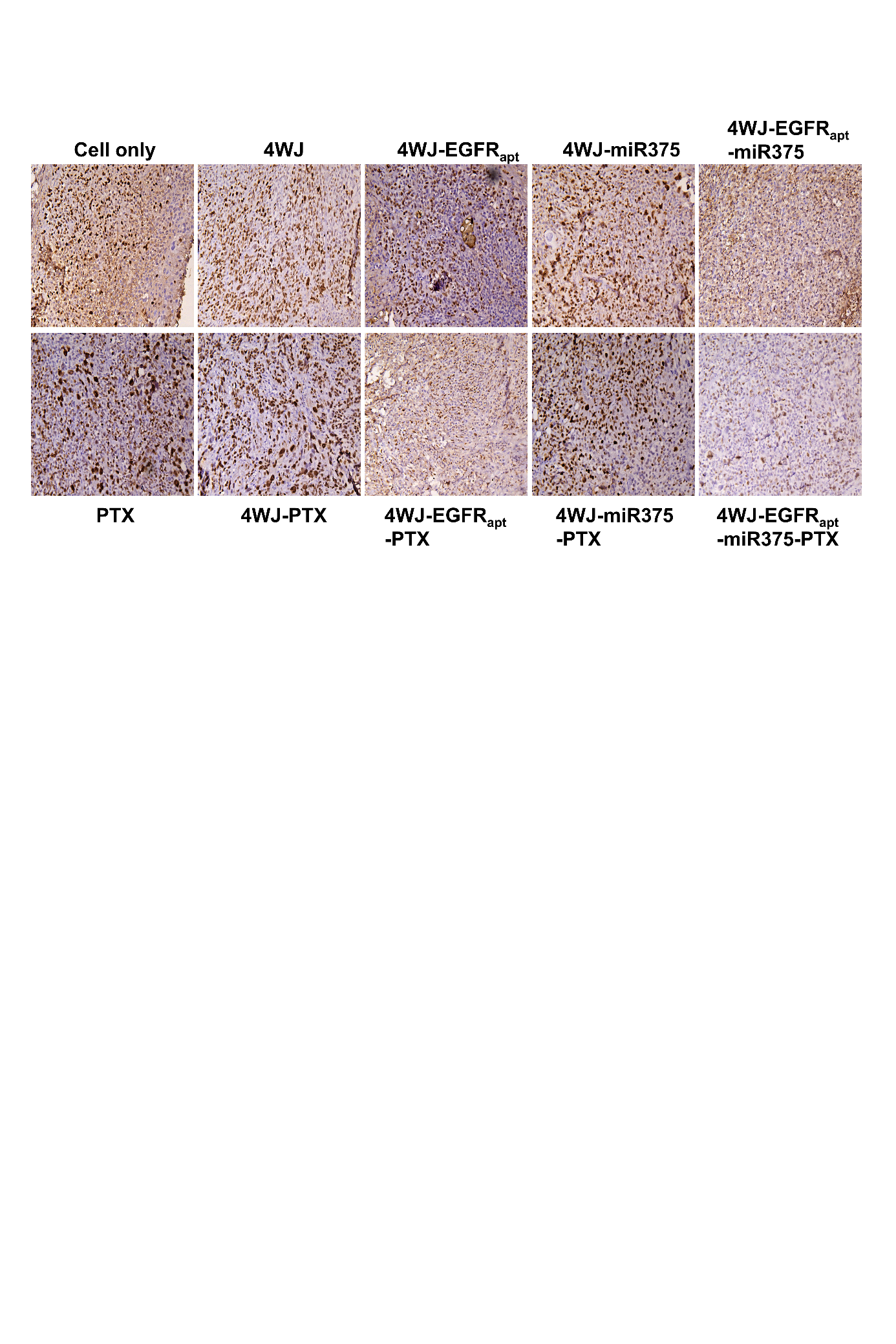


**Figure S13.** **Ki67 expression in tumor tissues.** KYSE-150 tumor-bearing mice were treated with PBS, 4WJ, 4WJ-EGFR_apt_, 4WJ-miR-375, 4WJ-EGFR_apt_-miR-375, PTX, 4WJ-PTX, 4WJ-EGFR_apt_-PTX, 4WJ-miR-375-PTX and 4WJ-EGFR_apt_-miR-375-PTX for 5 times, then Ki-67-positive cells (brown) in tumors were detected by immunohistochemistry. Scale bar: 100 μm.


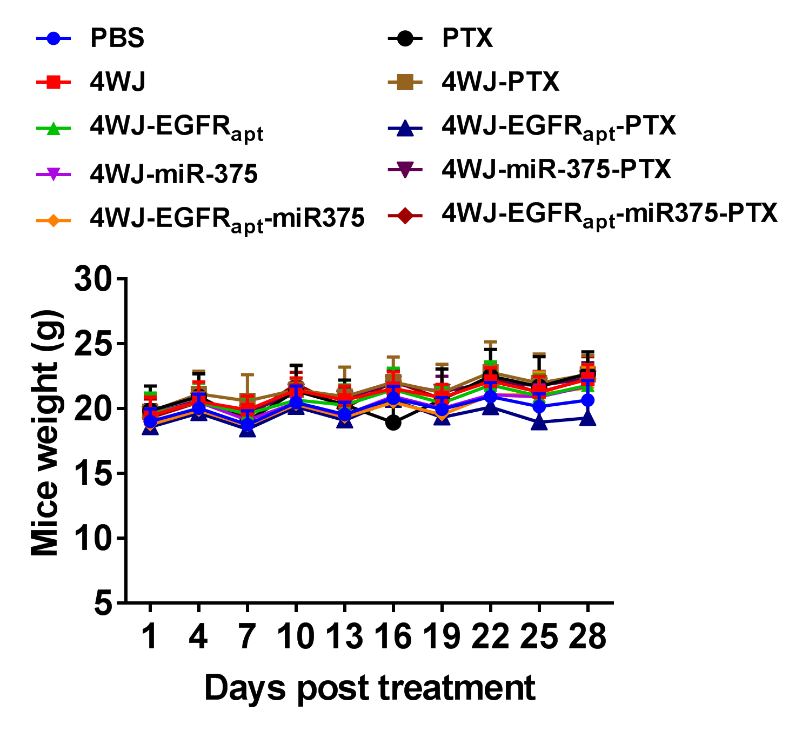


**Figure S14. Mice body weight changes.** Body weight of KYSE-150 tumor-bearing mice was measured every 3 days.


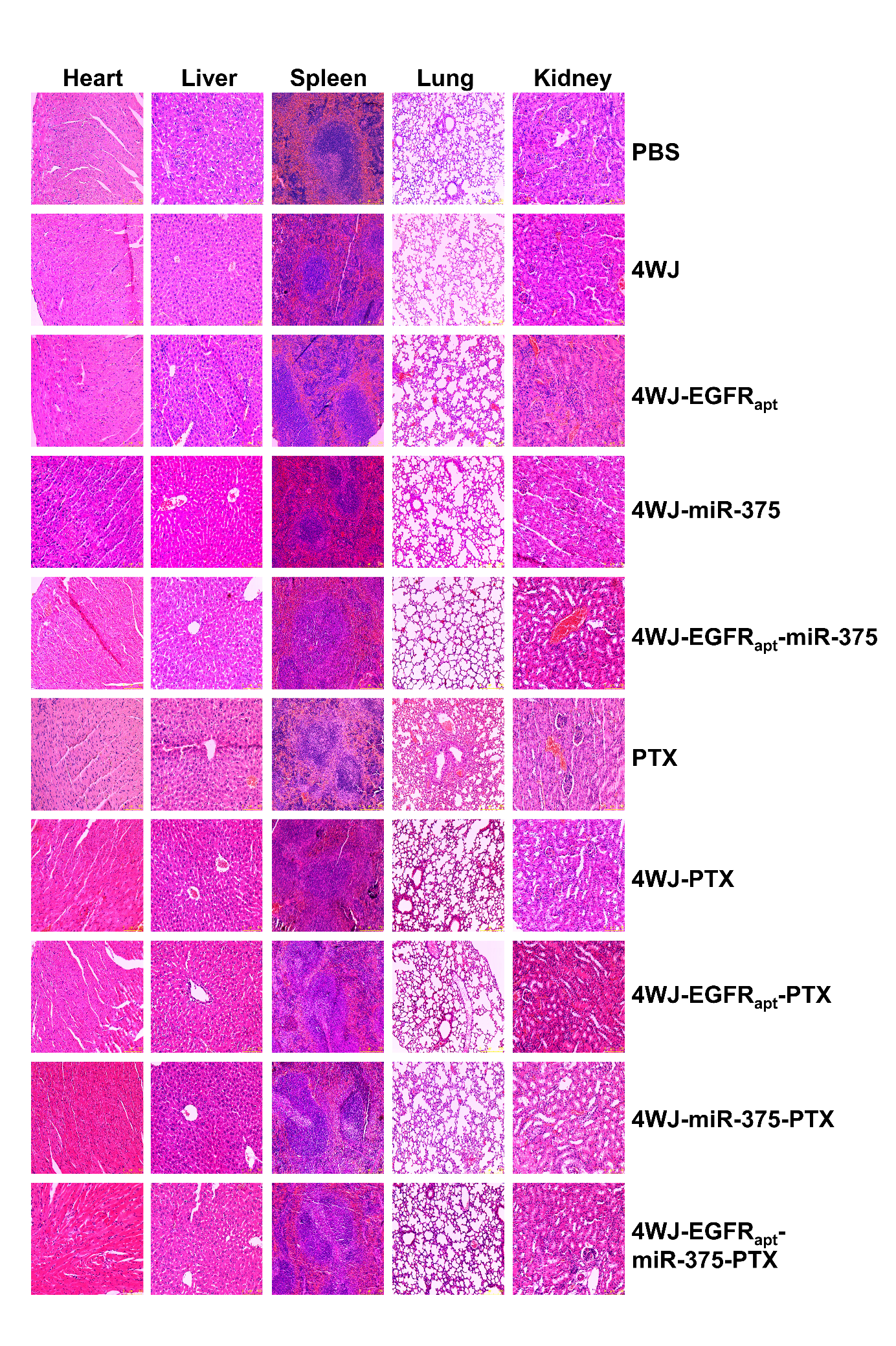


**Figure S15. Pathological changes of hearts, livers, spleens, lungs and kidneys was analyzed by HE staining.** KYSE-150 tumor-bearing mice were treated with PBS, 4WJ, 4WJ-EGFR_apt_, 4WJ-miR-375, 4WJ-EGFR_apt_-miR-375, PTX, 4WJ-PTX, 4WJ-EGFR_apt_-PTX, 4WJ-miR-375-PTX and 4WJ-EGFR_apt_-miR-375-PTX for 5 times, then the major organs were collected and the HE staning was performed to detect the pathological changes. Scale bar:100 μm.
